# Fluorescence tracking of inter- and intramolecular motion in zwitterionic aggregate

**DOI:** 10.1093/nsr/nwaf113

**Published:** 2025-04-01

**Authors:** Jin Wang, Zihao Deng, Xinwen Ou, Xinmeng Chen, Shengyi Yang, Jianyu Zhang, Xuan He, Jianwei Sun, Ryan T K Kwok, Jacky W Y Lam, Ben Zhong Tang

**Affiliations:** Department of Chemistry, Hong Kong Branch of Chinese National Engineering Research Center for Tissue Restoration and Reconstruction, Department of Chemical and Biological Engineering, Division of Life Science, and State Key Laboratory of Molecular Neuroscience, The Hong Kong University of Science and Technology, Hong Kong 999077, China; Department of Chemistry, Hong Kong Branch of Chinese National Engineering Research Center for Tissue Restoration and Reconstruction, Department of Chemical and Biological Engineering, Division of Life Science, and State Key Laboratory of Molecular Neuroscience, The Hong Kong University of Science and Technology, Hong Kong 999077, China; Department of Chemistry, Hong Kong Branch of Chinese National Engineering Research Center for Tissue Restoration and Reconstruction, Department of Chemical and Biological Engineering, Division of Life Science, and State Key Laboratory of Molecular Neuroscience, The Hong Kong University of Science and Technology, Hong Kong 999077, China; Department of Chemistry, Hong Kong Branch of Chinese National Engineering Research Center for Tissue Restoration and Reconstruction, Department of Chemical and Biological Engineering, Division of Life Science, and State Key Laboratory of Molecular Neuroscience, The Hong Kong University of Science and Technology, Hong Kong 999077, China; Department of Chemistry, Hong Kong Branch of Chinese National Engineering Research Center for Tissue Restoration and Reconstruction, Department of Chemical and Biological Engineering, Division of Life Science, and State Key Laboratory of Molecular Neuroscience, The Hong Kong University of Science and Technology, Hong Kong 999077, China; Department of Chemistry, Hong Kong Branch of Chinese National Engineering Research Center for Tissue Restoration and Reconstruction, Department of Chemical and Biological Engineering, Division of Life Science, and State Key Laboratory of Molecular Neuroscience, The Hong Kong University of Science and Technology, Hong Kong 999077, China; Department of Chemistry, Hong Kong Branch of Chinese National Engineering Research Center for Tissue Restoration and Reconstruction, Department of Chemical and Biological Engineering, Division of Life Science, and State Key Laboratory of Molecular Neuroscience, The Hong Kong University of Science and Technology, Hong Kong 999077, China; Department of Chemistry, Hong Kong Branch of Chinese National Engineering Research Center for Tissue Restoration and Reconstruction, Department of Chemical and Biological Engineering, Division of Life Science, and State Key Laboratory of Molecular Neuroscience, The Hong Kong University of Science and Technology, Hong Kong 999077, China; Department of Chemistry, Hong Kong Branch of Chinese National Engineering Research Center for Tissue Restoration and Reconstruction, Department of Chemical and Biological Engineering, Division of Life Science, and State Key Laboratory of Molecular Neuroscience, The Hong Kong University of Science and Technology, Hong Kong 999077, China; Department of Chemistry, Hong Kong Branch of Chinese National Engineering Research Center for Tissue Restoration and Reconstruction, Department of Chemical and Biological Engineering, Division of Life Science, and State Key Laboratory of Molecular Neuroscience, The Hong Kong University of Science and Technology, Hong Kong 999077, China; Department of Chemistry, Hong Kong Branch of Chinese National Engineering Research Center for Tissue Restoration and Reconstruction, Department of Chemical and Biological Engineering, Division of Life Science, and State Key Laboratory of Molecular Neuroscience, The Hong Kong University of Science and Technology, Hong Kong 999077, China; School of Science and Engineering, Shenzhen Institute of Aggregate Science and Technology, The Chinese University of Hong Kong, Shenzhen (CUHK-Shenzhen), Shenzhen 518172, China

**Keywords:** molecular dynamics, ionic aggregate, zwitterionic supramolecular polymer, self-recovery, ionic interaction

## Abstract

Ionic aggregates are among the most common forms of matter, yet the investigation of their molecular motion is often constrained by the instability of isolated anions and cations, as well as the lack of real-time monitoring techniques. This study presents a zwitterionic strategy that integrates both cations and anions into one fluorescent organic framework, forming a zwitterionic molecule. The zwitterionic strategy simplifies the intricate cation–anion systems that are typically found in conventional inorganic salts and imparts them with fluorescent properties, facilitating real-time tracking of ionic-interaction-induced molecular motion within ionic aggregates. Specifically, a blue shift in the fluorescence wavelength signified changes in aggregate states due to intermolecular motion, whereas a decrease in intensity was linked to intramolecular-motion-caused conformational changes. This spontaneous molecular motion enabled dynamic switching of the excited state energy-decay pathway, leading to switchable color–light responses. Overall, the zwitterionic strategy offers a novel framework for exploring the properties and behaviors of molecules in ionic aggregates.

## Teaser

A zwitterionic strategy integrates cations and anions into a single fluorescent framework, enabling real-time visualization of inter- and intramolecular motion through dynamic color-light switching in ionic aggregates.

## INTRODUCTION

In physical science, molecules are regarded as the fundamental units that embody the properties of matter [1–3] and are involved in continuous inter- and intramolecular motions [4–7]. Intermolecular motion pertains to the relative movement between different molecules whereas intramolecular motion refers to the relative movement of atoms within a molecule. The dynamics of molecular motion plays a crucial role in various processes, including thermal energy transfer [8,9], chemical reactions [10,11] and biological mechanisms [12–14]. For example, during DNA replication and transcription, the double-helix structure of the DNA molecule unwinds, enabling the internal rotations and intermolecular movements to facilitate the transcription of a single strand into RNA [15–17]. As a result, the study of molecular motion has emerged as a focal point of interest across multiple scientific disciplines. Aggregates represent the most common state of molecular existence [18–20]. It is generally believed that, when molecules transform from a solution or gas phase into an aggregate, their molecular motion is often suppressed. However, the reality is that molecules still undergo either vigorous or subtle inter- and intramolecular motions within aggregates [21,22], with dynamic crystals as one of the most representative examples [23,24].

**Figure 1.**
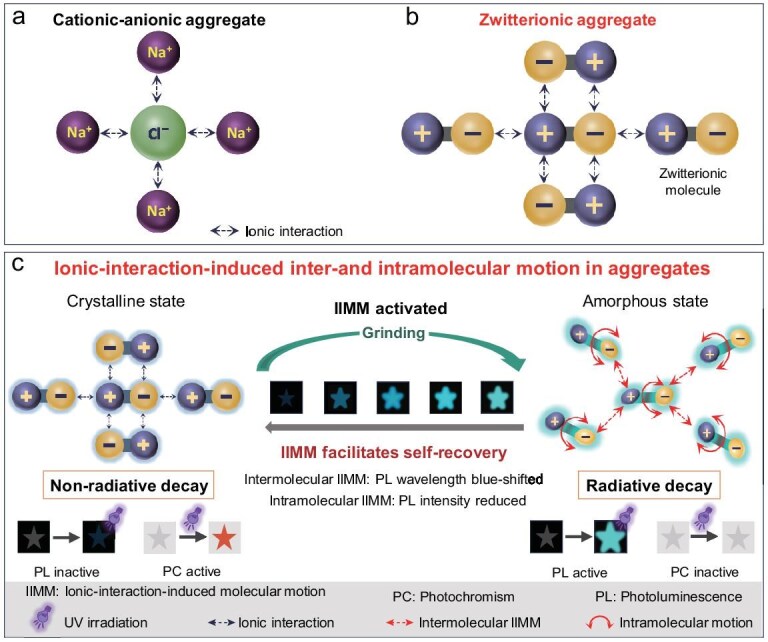
Fluorescence monitoring of inter- and intramolecular motion in zwitterionic aggregate. (a) Schematic diagram of aggregate structure in NaCl crystal. (b) Schematic diagram of chemical structure in zwitterionic aggregate. (c) Visualization of the inter- and intramolecular motion in zwitterionic aggregate through fluorescent signal.

Ionic aggregates are widely found in nature, industry and biological systems, with anions and cations considered to be the fundamental building units [25–27]. However, unlike molecules, it is difficult for ions to exist stably in isolation, which poses challenges for research. Additionally, unlike ordinary chemical bonds, ionic interactions lack directionality and saturation, further complicating the study [28,29]. Taking the simplest ionic aggregate, namely a sodium chloride (NaCl) crystal, as an example, one might consider the formula for NaCl as its fundamental building unit. However, NaCl crystals in fact do not contain discrete NaCl molecules. Instead, the crystal primarily consists of positively charged Na⁺ ions and negatively charged Cl^−^ ions that are arranged in a face-centered cubic structure. Thus, it seems more appropriate to regard Na⁺ and Cl⁻ ions as the fundamental building units (Fig. 1a). Nevertheless, anions and cations often coexist in the form of ion pairs, making it challenging for isolated cations and anions to exist stably in isolation. This presents difficulties in studying the structure and properties of individual ionic units. Moreover, the presence of two different types of ions further complicates the investigation [30,31]. As a result, research on the unit motion within ionic aggregates has been quite limited to date.

Due to the complexity of aggregate structures, understanding the dynamic nature of aggregates at a macroscopic level inevitably requires a means to relate molecular-level events to macroscopic transformations [23,32–34]. Photoluminescence (PL) is a fundamental optical property of molecules, with its intensity and wavelength closely related to the chemical structure [35,36]. Moreover, the same molecule in different conformations can exhibit varying PL signals at times, rendering PL a refined means for characterizing the conformational information of molecules in aggregates [37–39]. In recent years, our research group has successfully monitored molecular motion within aggregates by using fluorescence signals in different cases [40–42]. Given the complexity of traditional cation–anion aggregate structures, we have recently developed a zwitterionic strategy for simplifying them [43,44]. This approach clarifies the zwitterionic molecule as the fundamental unit for building ionic aggregates, thereby simplifying the intricate dual-unit systems that are typically observed in conventional inorganic salts. Furthermore, the zwitterionic strategy imparts fluorescent properties that are inherent to organic structures, offering a novel medium for real-time tracking of molecular motion within ionic aggregates (Fig. 1b). Specifically, this study first modified a tetraphenylethylene (TPE) backbone to obtain a zwitterionic molecule, namely 4,4′-(2,2-bis(4-ammoniophenyl)ethene-1,1-diyl)dibenzenesulfonate (TPE-2N2S), which contained both ammonium and sulfonate ions. Using this molecule as a building unit, a zwitterionic aggregate was obtained. Subsequently, ionic-interaction-induced molecule motion (IIMM) in the aggregate state was tracked via real-time monitoring changes in the PL. Notably, a blue-shifted wavelength corresponded to transition in aggregate states that was caused by intermolecular IIMM whereas a decrease in the PL intensity was associated with conformational changes due to intramolecular motion (Fig. 1c). Use of the zwitterionic molecule as a fundamental unit not only simplified the structure, but also combined the characteristics of ions and organic frameworks, making it possible to track both inter- and intramolecular motions within ionic aggregates.

## RESULTS AND DISCUSSION

### Tracking molecular motion with fluorescence

**Figure 2.**
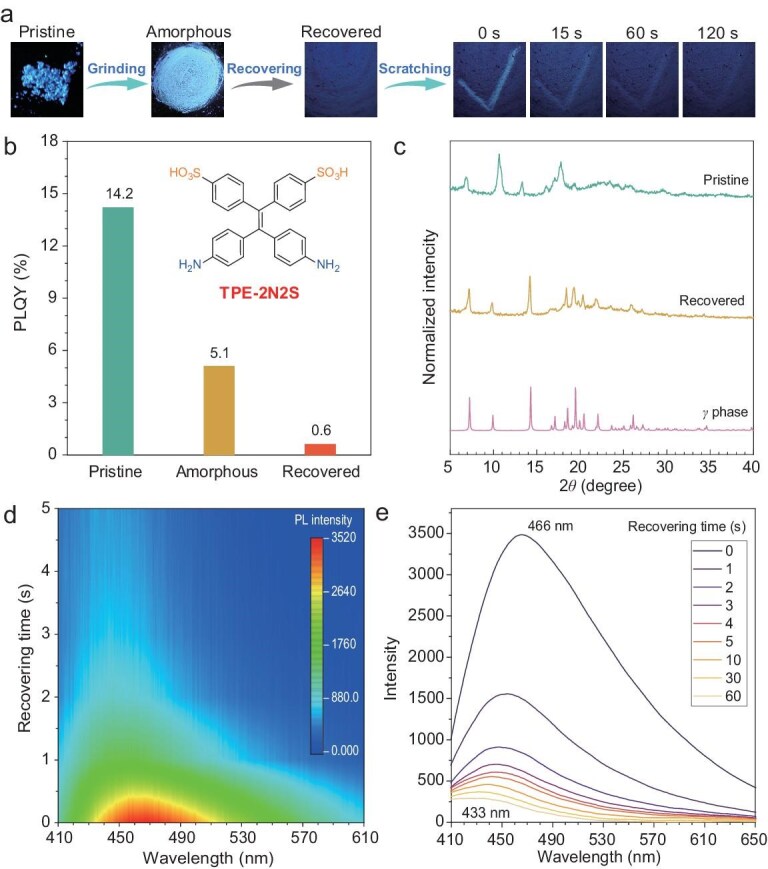
Tracking molecular motion in zwitterionic aggregate with PL signal. (a) Transition in PL signal of zwitterionic aggregate after grinding and self-recovery. (b) Changes in PLQY of zwitterionic aggregate after grinding and self-recovery. Inset: chemical structure of TPE-2N2S. (c) Changes in PXRD pattern after self-recovery. (d) and (e) Real-time tracking of changes in fluorescence spectra during self-recovery of zwitterionic aggregate after grinding ceased.

We first synthesized a zwitterionic molecule, namely TPE-2N2S, that featured both ammonium cations and sulfonate anions based on a TPE framework, forming a zwitterionic aggregate of pristine powder after aggregation from solution [43,44]. As shown in Fig. 2a and b, under ultraviolet (UV) light, the pristine powder of the TPE-2N2S emitted a bright deep-blue PL, with a PL quantum yield (PLQY) of 14.2%. Following subsequent grinding, the pristine powder was transformed into an amorphous state, resulting in a noticeable red shift in the PL, now appearing as a light-blue fluorescence, with the PLQY decreasing to 5.1%. Interestingly, upon removal of the grinding, the light-blue PL gradually faded and an almost invisible deep-blue fluorescence was eventually emitted. We designate these zwitterionic aggregates as three different forms: the pristine state (as prepared), the amorphous state (during grinding) and the recovered state (after grinding ceases), respectively.

To better observe the stimulus-responsive behavior, we applied a scratching action in the shape of a check mark on the recovered sample and the emergence of a light-blue fluorescent check mark at the corresponding location was observed. This mark exhibited significant fading after the grinding was ceased and ultimately returned to the recovered state after ∼120 seconds. As illustrated in Fig. 2d and e, real-time monitoring of the spontaneous transition of the PL after grinding removal by using fluorescence spectroscopy provided a more intuitive understanding of this dynamic behavior. Immediately upon removal of the grinding, the aggregate showed a strong PL at 466 nm. As the resting time increased, the PL noticeably diminished and blue-shifted to 433 nm. The transition of the PL during the grinding process indicated that the pristine powder, after being ground to the amorphous state and self-recovery, can spontaneously transition to a different aggregate form that distinguishes it from the pristine state—termed the recovered state. The transition and spontaneous recovery of the aggregate state after grinding suggested that TPE-2N2S retained molecular motion in aggregate. The powder X-ray diffraction (PXRD) pattern in Fig. 2c indicates that the crystalline structure of the recovered state after grinding transformed from the pristine state to the crystalline *γ* phase (CCDC number: 2339592) [43], further confirming the occurrence of molecular motion during the grinding and spontaneous recovering processes.

### IIMM in aggregate

**Figure 3.**
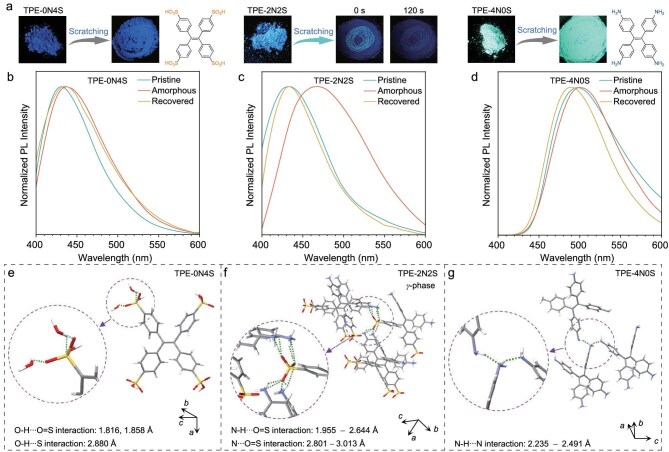
Comparative investigation of molecular motion activity and intermolecular interactions between aggregates with different building blocks. (a) Images depicting TPE-0N4S, TPE-2N2S and TPE-4N0S before and after exposure to scratching under UV light. Inset: chemical structures of TPE-0N4S and TPE-4N0S. (b–d) Fluorescence spectra for (b) TPE-0N4S, (c) TPE-2N2S and (d) TPE-4N0S are presented, illustrating the pristine, amorphous and recovered states. (e–g) Analysis of intermolecular interactions within the crystals of (e) TPE-0N4S, (f) TPE-2N2S and (g) TPE-4N0S.

Molecular motion is essential for the spontaneous recovery of the aggregate structure after grinding ceases. Previous studies have indicated that such recovery typically requires the introduction of intermolecular interactions, such as metal–π and π–π interactions, to drive spontaneous molecular motion in aggregate [40–42]. In our system, we consider whether similar interactions exist. Given the presence of both cations and anions in the TPE-2N2S molecule, it is likely that ionic interactions play a significant role in facilitating molecular motion within the aggregate. To verify this hypothesis, we compared the PL of three compounds: the zwitterionic TPE-2N2S, 4,4′,4″,4′″-(ethene-1,1,2,2-tetrayl)tetrabenzenesulfonic acid (TPE-0N4S, which contains only sulfonate groups), and 4,4′,4″,4′″-(ethene-1,1,2,2-tetrayl)tetraaniline (TPE-4N0S, which contains only amino groups) upon grinding. The results revealed that only TPE-2N2S, the zwitterionic compound, exhibited molecular motion activity in the aggregate. Interestingly, two similar zwitterionic compounds, namely the 4,4′,4″-(2-(4-ammoniophenyl)ethene-1,1,2-triyl)tribenzenesulfonate (TPE-1N3S) and 4-(1,2,2-tris(4-ammoniophenyl)vinyl)benzenesulfonate (TPE-3N1S), also exhibited PL activation and spontaneous decay upon grinding in the aggregate states, akin to TPE-2N2S (Figs S5−S9). In stark contrast, the TPE-0N4S bearing only sulfonate groups and TPE-4N0S with only amino groups did not demonstrate this dynamic process during grinding (Fig. 3a−d) and their corresponding PXRD patterns (Fig. S9) exhibited merely a trend from crystalline to amorphous states. These observations strongly suggested that ionic interactions were likely the driving force behind the spontaneous recovery behavior observed after grinding. Thus, the unique ionic characteristics of TPE-2N2S facilitated molecular motion, namely the IIMM, leading to its distinct photophysical properties compared with the other compounds.

To further validate the hypothesis regarding the role of ionic interactions, we examined the crystal structures of TPE-0N4S, TPE-2N2S and TPE-4N0S. As shown in Fig. 3e, TPE-0N4S in crystal revealed two types of intermolecular interactions: O−H···O=S and O−H···S interactions. In contrast, TPE-4N0S displayed only N−H···N interactions (Fig. 3g). The predominant interactions in the crystals of TPE-0N4S and TPE-4N0S were hydrogen-bonding interactions. The situation for TPE-2N2S was markedly different. Figure 3f illustrates the presence of strong ionic interactions between the sulfonate anions and ammonium cations in the TPE-2N2S crystal. These interactions were primarily characterized by N−H···O=S and N−H···S interactions, which were facilitated by the zwitterionic nature of TPE-2N2S. This distinct ionic character likely underpins the molecular motion observed in TPE-2N2S, further supporting the notion that IIMM is crucial for the spontaneous recovery after grinding ceases.

### Dynamic evolution in self-recovery

**Figure 4.**
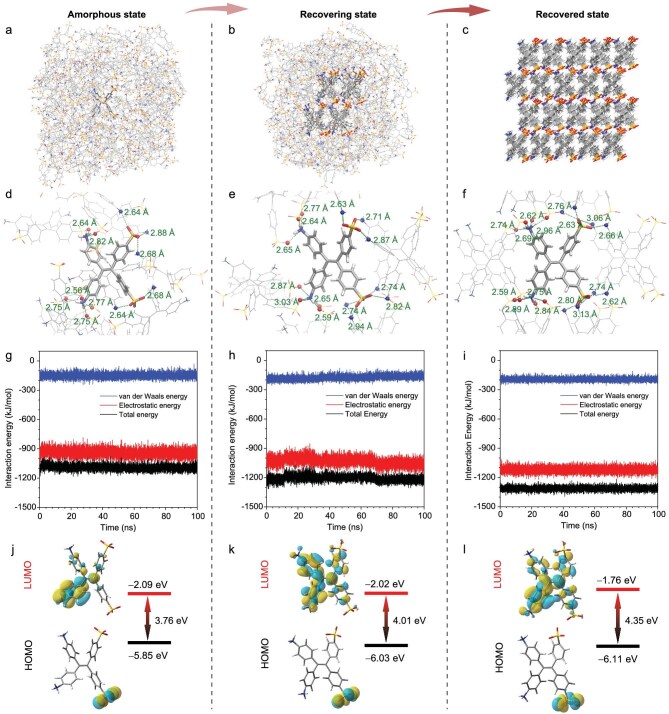
Theoretical investigation of molecular packing and photophysical characteristics of TPE-2N2S aggregates during the recovery process following grinding. (a–c) MD simulation models representing the (a) amorphous state, (b) recovering state and (c) recovered state. (d–f) Coordination environments for a selected molecule across these three aggregate states, where coordination is defined by the distance between an oxygen atom from the sulfonate group and a nitrogen atom from the ammonium group being <3.3 Å. (g–i) Interaction energies, comprising both Van der Waals and electrostatic components, between the representative molecule and surrounding molecules in the (g) amorphous state, (h) recovering state and (i) recovered state. (j–l) Frontier molecular orbitals for the representative molecules in the (j) amorphous state, (k) recovering state and (l) recovered state, derived from the optimized geometry of the amorphous state (S_0_). The aggregate optimization was performed by using an ONIOM model, with the optimized molecular structure in the quantum mechanics section extracted for single-point DFT calculations to elucidate electronic structure details.

To deepen our understanding of the self-recovery mechanism, comprehensive theoretical analyses were conducted by using molecular dynamics (MD) simulations alongside density functional theory (DFT) calculations. The initial phase involved MD simulations to examine the changes in the aggregate structure. Simultaneously, a representative TPE-2N2S molecule was selected to illustrate key features such as molecular stacking, local coordination environments and binding energies. As depicted in Fig. 4a, the grinding process disrupted the ordered crystalline arrangement and led to an amorphous phase, indicating the crystal stacking collapse in response to external stimuli. During this transition, the molecular configuration became entirely disordered, resulting in irregular coordination between the TPE-2N2S molecules and their neighbors. Notably, the two –SO_3_⁻ groups on the selected TPE-2N2S molecule interacted with two and three –NH_3_⁺ groups from adjacent molecules, respectively. The coordination numbers for these –NH_3_⁺ groups on the TPE-2N2S molecule were found to be 2 and 4, as shown in Fig. 4d. Additionally, this representative molecule exhibited electrostatic and Van der Waals energies of −939.72

and −150.81 kJ/mol, respectively, culminating in a total binding energy of −1090.53 kJ/mol. This value indicates a level of energetic instability within the amorphous state, as illustrated in Fig. 4g.

Upon removal of the grinding force, the system self-recovered toward the stable crystalline *γ* phase, facilitated by vigorous intermolecular IIMM. To model this recovering process, the aggregate structure of the recovering state was carefully constructed. In this configuration, a small cluster of molecules transformed back into the ordered crystalline *γ* phase whereas others remained in an amorphous state (Fig. 4b). In this phase, the –SO_3_⁻ groups of the representative TPE-2N2S molecule coordinated with three and four –NH_3_⁺ groups from adjacent TPE-2N2S molecules, respectively. Simultaneously, the two –NH_3_⁺ groups of the same TPE-2N2S molecule interacted with three and four –SO_3_⁻ groups from neighboring molecules (Fig. 4e). This increase in the coordination number indicated a strengthening of the ionic interactions between the TPE-2N2S molecules. Consequently, the electrostatic energy rose significantly, reaching −1030.66 kJ/mol. Additionally, the Van der Waals energy increased to −175.52 kJ/mol, resulting in an overall binding energy of −1206.18 kJ/mol, suggesting a transitional state between stability and instability (Fig. 4h).

As the coordination number increased and the binding energy strengthened, the vigorous intermolecular IIMM continued to facilitate the progression of the system toward its optimal recovered state (Fig. 4c). In this configuration, the ordered crystalline structure of the *γ* phase was reinstated and the local coordination environment of individual molecules became highly organized. Specifically, each –SO_3_⁻ group in the TPE-2N2S molecule coordinated with four –NH_3_⁺ groups from neighboring molecules, while each –NH_3_⁺ group similarly interacted with four –SO_3_⁻ groups, resulting in the highest binding energy observed among the three states during self-recovery (Fig. 4f). The electrostatic energy reached −1119.13 kJ/mol and the Van der Waals energy increased to −193.68 kJ/mol, culminating in a total binding energy of −1312.81 kJ/mol (Fig. 4i). This elevated binding energy indicated a significant enhancement in stability within the recovered state. The simulations of the aggregate structures effectively illustrated the transition from the *γ* phase to the amorphous state that was induced by grinding, as well as the subsequent recovery that was driven by ionic interactions. Notably, the variations in the molecular coordination numbers and the resulting ionic interactions during grinding appear to be key factors that influence the self-recovery of the aggregate states and their associated photophysical properties.

### Intermolecular motion facilitated blue-shifting in PL wavelength

In addition to the alterations in the molecular arrangement and binding energy that resulted from the grinding and self-recovery, changes in the molecular conformation and the corresponding electronic structure were examined by using DFT calculations. This investigation aimed to clarify the mechanism behind the observed PL wavelength shifts of TPE-2N2S upon grinding and self-recovery. As previously noted, grinding activated the PL of TPE-2N2S, causing the emission peak to shift from 433 nm (associated with the recovered state) to 466 nm, accompanied by increased intensity. This shift ultimately reverted to the recovered state, in which the emission intensity was negligible. The molecular conformation was influenced by the coordination environment; thus, the geometries of the amorphous, recovering and recovered states were derived from their respective MD simulation structures and optimized by using our own N-layered integrated molecular orbital and molecular mechanics (ONIOM) model based on the quantum mechanics (QM)/molecular mechanics (MM) method.

Following optimization, the molecule extracted from the QM portion of the ONIOM model was analysed to elucidate the electronic structure and explain the observed red shift in the PL spectra. As shown in Fig. 4j–l, the transition of the zwitterionic aggregate from the amorphous state to the recovering state and finally to the recovered state was accompanied by a gradual decrease in the highest occupied molecular orbital (HOMO) levels of TPE-2N2S from −5.85 to −6.03 eV, and then to −6.11 eV. Concurrently, the lowest unoccupied molecular orbital (LUMO) levels increased from −2.09 to −2.02 eV, and finally to −1.76 eV. A lower HOMO level indicated reduced electron-loss potential, reflecting greater donor stability, whereas a higher LUMO level suggested decreased electron-gain potential, indicating enhanced acceptor stability. The observed trends in the HOMO and LUMO levels upon the cessation of grinding indicated a strengthening of the coordination interactions between the –SO_3_⁻ and –NH_3_⁺ groups of TPE-2N2S and surrounding molecules, thereby enhancing molecular stability. This coordination and structural evolution resulted in an increase in the HOMO–LUMO gap from 3.76 eV in the amorphous state to 4.01 eV in the recovering state, reaching 4.35 eV in the recovered state. The smaller HOMO–LUMO gap in the amorphous aggregate was consistent with longer emission wavelengths compared with the recovered state, aligning with experimental observations. The intermolecular ionic interactions and motions promoted a transition from disordered to crystalline states that specifically manifested as changes in the ion coordination number and molecular arrangement at the microscopic level, resulting in the blue shift in the PL during self-recovery.

### Intramolecular motion facilitated fading in PL intensity

**Figure 5.**
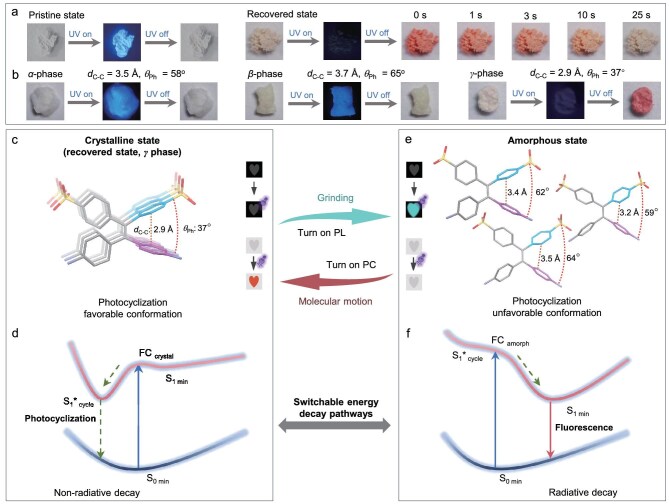
Investigation of the mechanism of fading in PL intensity following grinding. (a) Images of TPE-2N2S observed under UV light and in daylight following UV irradiation. (b) Images of TPE-2N2S in crystalline α, β and *γ* phases under UV light, daylight and daylight immediately after UV irradiation. (c) Depiction of the uniform molecular packing of TPE-2N2S in the crystalline *γ* phase, highlighting a conformation conducive to photocyclization. Inset: demonstration of PL inactivity alongside PC activity in the crystalline *γ* phase. (d) Schematic representation of the non-radiative decay pathway resulting from the photocyclization reaction upon UV irradiation. (e) Illustration of disordered packing of TPE-2N2S exhibiting conformations that are unfavorable for photocyclization in the amorphous state. Inset: evidence of PL activity while PC activity is absent in the amorphous state. (f) Schematic representation of the radiative decay pathway resulting from suppressed photocyclization reactivity upon UV irradiation.

Fluorescence, the fundamental behavior of PL, arises from the rapid release of energy through radiative decay following light absorption. When the recovered powder was subjected to strong UV light in daylight, it transformed from a pale yellow to red (Fig. 5a), reverting to its original color after 25 seconds of UV light removal, demonstrating photochromic (PC) activity (Fig. S10). This phenomenon is commonly attributed to photoactivated reactions, which facilitate the non-radiative decay of excited states [45–47]. In diarylethylene derivatives, photocyclization reactions are believed to drive the PC behavior and the efficacy of the reaction is closely linked to the molecular conformation [48,49]. Specifically, a shorter C–C distance (*d*_C–C_) between potential cyclization sites and a favorable dihedral angle (*θ*_Ph_) between adjacent phenyl rings enhance photocyclization efficiency. As illustrated in Fig. 5b, the larger *d*_C–C_ and *θ*_Ph_ values in crystals *α* (CCDC number: 2339593) and *β* inhibited photocyclization upon UV exposure [43], resulting in inactivity regarding PC activity (Figs S11−S15). In these crystals, the excited state energy could not be released through non-radiative photocyclization, enabling PL activity to manifest. Conversely, in crystal *γ*, the *d*_C–C_ and *θ*_Ph_ values measured 2.9 Å and 37°, respectively, allowing photocyclization to occur under UV irradiation. The products of this reaction showed significant absorption at 510 nm, confirming PC activity in crystal *γ*. Consequently, the dissipation of the excited state energy via photocyclization suppressed the corresponding radiative decay pathway, leading to diminished PL performance in crystal *γ*.

TPE molecules exhibit conformational repulsion between adjacent phenyl rings, favoring a *θ*_Ph_ value of between 50° and 65° and a corresponding *d*_C–C_ value of >3.2 Å. As shown in Fig. 5c, in the crystalline *γ* phase, TPE-2N2S molecules demonstrated a well-organized arrangement with a *d*_C–C_ value of 2.9 Å and a *θ*_Ph_ value of 37°, which supported a conformation that was conducive to photocyclization. Strong ionic interactions effectively counteracted the conformational repulsion, resulting in this confined structure of TPE-2N2S in crystal *γ*. Upon UV exposure, the molecules transitioned from the ground state (S_0_) to the excited state (S_1_), favoring energy dissipation through the non-radiative decay pathway associated with the reversible photocyclization reaction, ultimately returning to the ground state (Fig. 5d). In addition to quenching PL, the photocyclization reaction produced a cyclized intermediate with a prominent absorption peak at 510 nm, indicating PC activity. This reaction was reversible, allowing the cyclized form to revert to the original open-ring state. In the amorphous state, TPE-2N2S molecules exhibited reduced intermolecular ionic interactions due to the disruption of the crystalline structure. This suggested a partial relaxation of the constraints that were imposed by strong ionic interactions in crystal *γ*, facilitating a transition to less-constrained conformations with increased *θ*_Ph_ and *d*_C–C_ values. By analysing the single-molecule structure that was extracted from the simulated amorphous state in Fig. 4a, the resulting conformations of TPE-2N2S could be elucidated (Fig. 5e), displaying higher *d*_C–C_ and *θ*_Ph_ values that were unfavorable for photocyclization. Consequently, upon UV irradiation, these molecules more readily dissipated energy through the radiative fluorescence pathway, returning to the ground state (Fig. 5f). These conformational changes after grinding had ceased transformed the photocyclization capability of TPE-2N2S from an inactive state in the amorphous phase to an active state in the crystalline *γ* phase, with intramolecular motion during the recovery of ground powder leading to a sudden reduction in the PL intensity. Overall, the fluorescence signal has been demonstrated to be an effective tool for linking the dynamic characteristics of aggregates at molecular and macroscopic levels [23,50].

### IIMM facilitated dynamic switching in color and light activity

Grinding induced a transition in TPE-2N2S molecules from a conformation that was conducive to photocyclization in the crystalline *γ* phase to one that was unfavorable in the amorphous state. This change altered the energy dissipation pathway in the excited state, shifting it from non-radiative decay to radiative decay, which activated PL while suppressing PC activity. Upon the cessation of grinding, IIMM in aggregates facilitated the self-recovery of the system back to the crystalline *γ* phase. This transition retained the non-radiative decay pathway associated with photocyclization, reactivating PC while deactivating PL. Overall, IIMM enabled dynamic switching between radiative and non-radiative decay pathways, prompting further investigation into the multimodal stimuli-responsive behaviors that are related to light and color changes.

**Figure 6.**
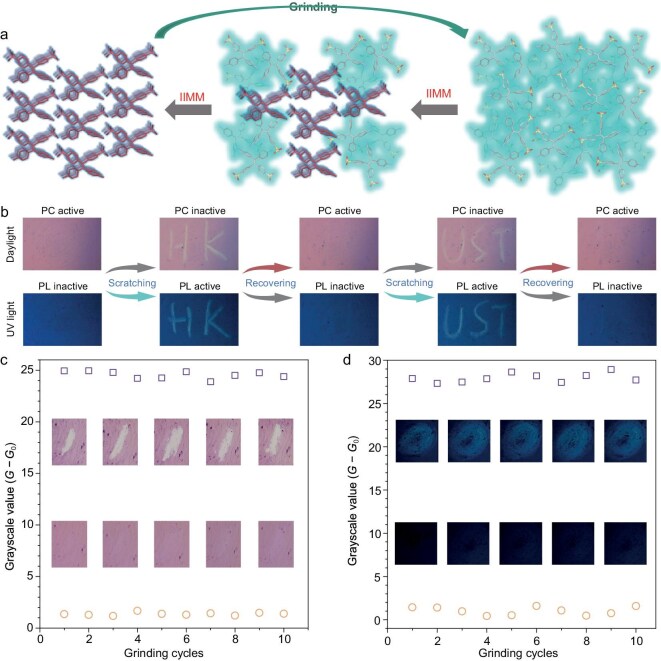
Dynamic switching between PC and PL activity in zwitterionic aggregate. (a) Schematic diagram illustrating the changes in PC and PL activity corresponding to transitions in aggregate states. (b) Dynamic switching observed between PC and PL activity during the scratching and recovery processes, with monitoring focused exclusively on the scratched regions to assess PC and PL activities. (c) Variations in grayscale values during the activation and deactivation of PC activity via grinding and subsequent self-recovery over 10 cycles. (d) Variations in grayscale values during the activation and deactivation of PL activity through grinding and self-recovery for 10 cycles. The parameter *G* − *G*_0_ denotes the difference in greyscale values before and after grinding, where *G* represents the value immediately following grinding and *G*_0_ indicates the value prior to grinding.

As illustrated in Fig. 6a and b, significant PC activity was observed in the recovered state under daylight after UV irradiation whereas the PL remained negligible. When scratches shaped like ‘HK’ were introduced, the letters became visible in daylight following UV exposure, indicating a loss of PC activity in the scratched regions. However, PL activity was activated under UV light. After 120 seconds of self-recovery that was facilitated by IIMM, the scratches faded, signifying the reactivation of PC activity and the suppression of PL. Subsequent scratches in the shape of ‘UST’ resulted in the visibility of these letters in both daylight and UV light, reflecting the suppression of PC activity while PL was reactivated in the scratched areas. Similarly, after another 120 seconds, the letters gradually disappeared. Thus, TPE-2N2S transitioned from a recovered state that was characterized by active PC and inactive PL to an amorphous state with activated PL and suppressed PC. Upon cessation of scratching, the system spontaneously reverted to the recovered state, restoring PC activity while PL became inactive. This phenomenon was demonstrated to be repeatable across multiple cycles. As shown in Fig. 6c, during 10 cycles of scratching and recovery, TPE-2N2S successfully switched between active and inactive states for PC. In contrast, Fig. 6d illustrates the remarkable switching capability of TPE-2N2S between inactive and active states for PL. The dynamic transitions of PC and PL activities that were induced by scratching and subsequent self-recovery through inter- and intramolecular motion suggest that this mechanical action enables convenient and repeatable switching between color and light activities.

## CONCLUSION

The dynamics of molecular motion are pivotal in numerous processes. The aggregate state represents the most prevalent form of matter; however, the inherent complexity of aggregate structures poses significant challenges for monitoring molecular motion within them. Ionic aggregates, which typically comprise both cations and anions, further complicate this structure due to their dual-unit system. The challenge of capturing and studying ions that exist stably in isolation continues to hinder real-time monitoring of molecular motion within ionic aggregates. In this study, we successfully incorporated both cations and anions into a fluorescent TPE backbone, resulting in a zwitterionic molecule, namely TPE-2N2S. This zwitterionic molecule clarifies the fundamental unit for constructing ionic aggregate, thereby simplifying the complex cation–anion dual-unit systems that are commonly encountered in conventional inorganic salts. Additionally, this approach endows the ions with the intrinsic fluorescent properties of the organic structure, facilitating real-time tracking of molecular motion within ionic aggregates.

By utilizing this strategy, we achieved real-time monitoring of both inter- and intramolecular motion within an ionic aggregate by tracking changes in the PL wavelength and intensity of the TPE-2N2S aggregate. Specifically, following mechanical grinding, intermolecular IIMM induced a transition of the aggregate structure from an amorphous to a crystalline state, resulting in a blue shift of the PL wavelength. Meanwhile, intramolecular motion led to a transformation in the molecular conformation of TPE-2N2S from photocyclization inactive to an active conformation, causing a shift in the excited state energy-decay pathway from radiative to non-radiative decay. This transition quenched PL while simultaneously activating PC activity. The dynamic switching of the energy-decay pathway, driven by IIMM, enabled the activation of multimodal photonic responses, characterized by switchable color–light responses in the aggregate. The zwitterionic strategy presents a novel approach for investigating the properties and behaviors of ionic molecules in the aggregate state, significantly advancing our understanding of ionic aggregates.

## METHODS

### MD simulations

To model the amorphous aggregate, 160 molecules were randomly positioned within a cubic box measuring 6 nm on each side. Energy minimization was carried out by using the steepest descent algorithm to relax the system. The initial conformation was then derived from a 50-ns NPT (constant number of particles, constant pressure, and constant temperature) ensemble simulation at 1 atm and 300 K. This final conformation was subsequently transferred to a larger box with a side length of 18 nm. The aggregate configuration underwent equilibration through a 50-ns NVT (constant number of particles, constant volume, and constant temperature) ensemble simulation at 400 K. Following equilibration, a 100-ns production run in the NVT ensemble at 400 K was performed to gather data.

To simulate the recovering process of the amorphous aggregate into a stable crystal, a small cluster of 16 molecules (seed crystal) was extracted from the equilibrated crystal structure and positioned in a 6-nm cubic box. Surrounding this seed crystal, 144 additional molecules were randomly placed. Energy minimization was performed by using the steepest descent algorithm to relax the surrounding molecules. The initial conformation was obtained from a 50-ns NPT ensemble simulation at 1 atm and 300 K, during which position restraints were applied to the atoms of the seed crystal by using a harmonic potential of 10 000 kJ mol⁻¹ nm⁻². The final conformation was then transferred to a larger box with an 18-nm side length. Prior to the production run, further equilibration was conducted in three steps. In the first step, position restraints were applied to the seed crystal atoms with a harmonic potential of 10 000 kJ mol⁻¹ nm⁻², followed by a 50-ns NVT ensemble simulation at 300 K. In the second step, the restraint force on the seed crystal atoms was reduced to 1000 kJ mol⁻¹ nm⁻² and the system underwent another 50-ns NVT ensemble simulation at 300 K. In the final step, all position restraints were removed, allowing all atoms to move freely during a subsequent 50-ns NVT ensemble simulation at 300 K for complete equilibration. After equilibration, a 100-ns production run in the NVT ensemble at 300 K was conducted to collect data.

To model the stable crystal, a cluster of 160 molecules was extracted from the crystal packing structure and placed in a box measuring 5.466 20 nm × 3.710 48 nm × 4.875 80 nm. The system underwent an equilibration process that included energy minimization using the steepest descent algorithm, followed by a 50-ns NVT ensemble simulation at 200 K. After equilibration, a 100-ns production run in the NVT ensemble at 200 K was performed to gather data. The force-field parameters for the TPE-2N2S molecule were obtained from the CHARMM General Force Field. Temperature and pressure control were achieved by using the V-rescale thermostat and the Berendsen barostat, respectively. A cut-off distance of 1.2 nm was applied for the calculation of short-range electrostatic and Van der Waals interactions. Long-range electrostatic interactions were treated by using the particle mesh Ewald method. The LINCS algorithm was utilized to constrain bonds that involved hydrogen atoms. Periodic boundary conditions were applied in all three dimensions. All MD simulations were performed by using the GROMACS 2020 package.

### Quantum chemical calculations

The initial geometries for the amorphous, recovering and recovered states were derived from their respective MD simulation structures and then optimized by using an ONIOM model that combines QM and MM methods. A representative molecule was selected as the QM component and optimized via DFT at the M06-2X-D3/6–31G(d, p) level. The surrounding molecules were treated as the MM component, fixed in place and described by using the universal force field. Atomic charges were assigned by utilizing the restrained electrostatic potential model based on the M06-2X-D3/6–31G(d, p) level. Frequency calculations were conducted at the same level of theory to confirm that the optimized structure represented a minimum on the potential energy surface. The optimized QM molecule was subsequently extracted from the ONIOM model for additional single-point DFT calculations. All quantum chemical calculations were performed by using the Gaussian 16 software package (see Refs S1–S10 in supporting information).

## Supplementary Material

nwaf113_Supplemental_Files

## Data Availability

CCDC 2339593, 2354896, 2339592, 2355139 and 2354897 contain the supplementary crystallographic data for this paper. These data can be obtained free of charge via www.ccdc.cam.ac.uk/data_request/cif, by emailing data_request@ccdc.cam.ac.uk or by contacting The Cambridge Crystallographic Data Centre, 12 Union Road, Cambridge CB2 1EZ, UK; fax: +44 1223 336033.
