# Supplementary material for: Fluorescence tracking of inter- and intramolecular motion in zwitterionic aggregate
Source: Natl Sci Rev. 2025 Apr 1;12(6):nwaf113. doi: 10.1093/nsr/nwaf113 (PMC12051867; doi:10.1093/nsr/nwaf113)
Supplement: nwaf113_Supplemental_Files [file nwaf113_supplemental_files.zip › Supplementary information-revised.pdf]

Supporting information

Fluorescence Tracking of Inter- and Intramolecular Motion in Zwitterionic Aggregate

Jin Wang, Zihao Deng, Xinwen Ou, Xinmeng Chen, Shengyi Yang, Jianyu Zhang, Xuan He, Jianwei Sun, Ryan T. K. Kwok*, Jacky W. Y. Lam* and Ben Zhong Tang*

1. Materials

Tetraphenylethylene (TPE, 97%), acetic acid (99%), concentrated nitric acid (70%), hydrazine hydrate (97%), palladium on carbon (10%), concentrated sulfuric acid (95%). All the above-mentioned chemicals were purchased from Energy Chemistry, Sigma-Aldrich, and TCI, and used directly without further purification. All the solvents were purchased from VWR Chemicals Corp.

2. Synthesis

TPE-1N, TPE-2N, TPE-3N, and TPE-4N0S: 0.5 g TPE was dissolved in 20 mL DCM, and 12 mL Acetic acid was then added drop wise under stirring. 10 mL concentrated Nitric acid was dropwise added at 0 ^{o}C, then moved into RT and kept for 3 h to finish the reaction. The reactant was pour into 100 mL water, and purified by column chromatography on silica gel with DCM-hexane (2:3, v/v) as eluent to get (2-(4-nitrophenyl)ethene-1,1,2-triyl)tribenzene (yield: 90%). Similarly, the (Z)-1,2-bis(4-nitrophenyl)-1,2-diphenylethene, 4,4',4"-(2-phenylethene-1,1,2-triyl)tris(nitrobenzene), and 1,1,2,2-tetrakis(4-nitrophenyl)ethene was prepared with increased Acetic acid and concentrated Nitric acid content (yield: 70-95%). After that, these products were subjected to reduction reactions. 0.5 g (Z)-1,2-bis(4-nitrophenyl)-1,2-diphenylethene and 0.01 g Palladium on carbon was dissolved in 5 mL THF, and 5 mL Hydrazine hydrate was added. under N_2 atmosphere, the reactant was kept in 70 ^{o}C oil bath overnight. The crude product was filtered and washed with THF, and finally removing the solvent in vacuo to obtain TPE-1N (yield: 90%). TPE-2N, TPE-3N, and TPE-4N0S was synthesized with the same procedure, except that the TPE-4N0S was purified by column chromatography on silica gel with EA-hexane (1:2, v/v) as eluent (yield: 85-95%).

TPE-0N4S, TPE-1N3S, TPE-2N2S, and TPE-3N1S: 0.5 g TPE was added with 5 mL sulfuric ccid and stirred for 3h at 110 ^{o}C. The reaction mixture was poured into 100 mL EA and filtered to get white powder of TPE-0N4S with a yield of 98% without further purification. TPE-1N3S, TPE-2N2S, and TPE-3N1S was synthesized from TPE-1N. TPE-2N, and TPE-3N with similar procedure (yield: 95-97%), respectively.

Culture of crystals: 1 mg TPE-2N2S was dissolved in 0.5 mL sulfuric acid, and 1ml water was put onto the acid phase. The crystal-γ was culture after 3 days. The crystal-α was cultured by replacement of water by methanol. 1 mg TPE-2N2S was dissolved in 5 mL DMSO and filtered, then, 1 mL EA was put onto the DMSO phase. The crystal-β was culture after 2 days. 1 mg TPE-0N4S was dissolved in 1 mL methanol as good solvent, and the crystal was cultured with permeation method through bad solvent volatilization (EA). 1 mg TPE-4N0S was dissolved in 1 mL THF, the crystal was cultured by solvent volatilization.

3. Methods

Characterization. Nuclear magnetic resonance (NMR) spectra were tested on a Bruker AVIII 400 MHz NMR spectrometer equipped with a Dual Probe. Chemical shifts (δ) are given in ppm relative to TMS. The residual solvent signals were used as references and the chemical shifts were converted to the TMS scale. High-resolution mass spectra (HRMS) were estimated on a GCT premier CAB048 mass spectrometer. The photoluminescence (PL) spectra were recorded on a Horiba Fluorolog-3 spectrofluorometer. UV-vis reflectance spectra were recorded on an Ocean Optic QE65 Pro spectrometer with the reflection probe R600-125F. UV light source for the irradiation process was Ocean Optic D-2000 deuterium lamp. The photoluminescence quantum yield (PLQY) was collected by an integrating sphere on Hamamatsu Quantum Yield Spectrometer C11347 Quantaurus. Powder X-ray diffraction (PXRD) data were collected on a Rigaku Ultima-IV automated diffraction system using Cu Kα emission radiation (λ = 1.5406 Å) at room temperature in a range of 5-50° (2θ) with a scan speed of 2°/min and the operating power was 40 kV/44 mA. Single crystal data were collected on a SuperNova, Dual, Cu at home/near, Atlas diffractometer. Using Olex2, the structure was solved with the SHELXT structure solution program using Intrinsic Phasing and refined with the SHELXL refinement package using Least Squares minimization. CCDC 2339593, 2354896 and 2339592 contain the supplementary crystallographic data for crystal-α, β, and γ of TPE-2N2S, respectively. CCDC 2355139 and 2354897 contain the supplementary crystallographic data for crystal of TPE-0N4S and TPE-4N0S, respectively. All digital photos were recorded on a Canon EOS 60D camera. DFT calculations were conducted on

CrystalExplorer program at the B3LYP/6-31G (d, p) level. The structures were extracted from single crystal structures directly without any optimization.

Dynamic switching. The pristine powder of TPE-2N2S (0.2 g) was placed on an agate mortar. The grinding force was applied to transform pristine powder into ground powder. Upon removing grinding, the ground powder spontaneously transformed to the recovered state. Further grinding and recovering realized the dynamic switching between ground state and recovered state, accompanied by the on/off of PL and PC activity. The images for illustrating dynamic switching between color and light was taken in daylight with weak UV, or in weak UV light.

4. Computational details

Molecular dynamics simulations

To simulate the amorphous aggregate, 160 molecules were randomly placed in a cubic box with a side length of 6 nm, and the energy minimization using the steepest descent algorithm was performed to relax the system. The initial conformation was then obtained from a 50 ns *NPT* ensemble simulation (P = 1 atm and T = 300 K). The final conformation was subsequently placed into a big box with a side length of 18 nm. The aggregate configuration was equilibrated by a 50 ns *NVT* ensemble simulation (T = 400 K). Following the equilibration, a 100 ns production run in *NVT* ensemble (T = 400 K) was conducted to collect data.

To simulate the recovering process of the amorphous aggregate to the stable crystal, a small cluster of 16 molecules (seed crystal) were extracted from the equilibrated crystal structure and placed in a small box with a side length of 6 nm. Additionally, 144 molecules were randomly positioned around the seed crystal. The energy minimization using steepest descent algorithm was used to relax the surrounding molecules. The initial conformation was then obtained from a 50 ns *NPT* ensemble simulation (P = 1 atm and T = 300 K), in which the position restraints were applied to the atoms of the seed crystal using a harmonic potential of 10000 kJ mol^{-1} nm^{-2}. The final conformation was then placed into a big box with a side length of 18 nm. Prior to the production run, further equilibration was performed in three steps. In the first step, position restraints were applied to the atoms of the seed crystal with a harmonic potential of 10000 kJ mol^{-1} nm^{-2}, and the system underwent *NVT* ensemble simulation (T = 300 K) for 50 ns. In the second step, the restrained force on the atoms of the seed crystal was reduced to 1000 kJ mol^{-1} nm^{-2}, and the system was subjected to another 50 ns *NVT* ensemble simulation (T = 300 K). In the third step, all the position restraints were removed, allowing all atoms in the system to move freely during a 50 ns NVT ensemble simulation (T = 300 K) for full equilibration. After the equilibration, a 100 ns production run in *NVT* ensemble (T = 300 K) was performed to collect data.

To simulate the stable crystal, a cluster containing 160 molecules was extracted from the crystal packing structure and placed in a compatible box with the size of 5.46620 nm × 3.71048 nm × 4.87580 nm. The system underwent an equilibration procedure that included the energy minimization using the steepest descent algorithm, followed by a 50 ns *NVT* ensemble simulation (T = 200 K). After the equilibration, a 100 ns production run in *NVT* ensemble (T = 200 K) was conducted to collect data.

The force field parameters of TPE-2N2S molecule were taken from CHARMM General Force Field (CGenFF) [1-2]. The system temperature and pressure were controlled using the V-rescale thermostat [3] and the Berendsen barostat [4], respectively. A typical cutoff distance of 1.2 nm was applied for calculating the short-range electrostatic interactions and the Van der Waals interactions. The particle mesh Ewald (PME) method was employed for the long-range electrostatic interactions.[5-6] The LINCS algorithm was adopted to constrain the bonds involving hydrogen atoms.[7] The periodic boundary conditions (PBC) were applied in all three dimensions.[8] All the molecular dynamics (MD) simulations were carried out using the GROMACS 2020 package.[9]

Quantum chemical calculations

The initial geometries of the ground state, recovering state, and recovered state were derived from their respective MD simulation structures, and subsequently optimized using an ONIOM model that combines quantum mechanics (QM) and molecular mechanics (MM) methods. One representative molecule was selected as the QM part and optimized using density functional theory (DFT) at the M06-2X-D3/6-31G(d,p) level. The surrounding molecules were frozen to act as the MM part with the universal force field (UFF), and the restrained electrostatic potential (RESP) model based on M06-2X-D3/6-31G(d,p) level was used to assign the atomic charges. The frequency calculations were carried out at the same level of theory to confirm that the optimized structure corresponds to a minimum point on the potential energy surface. Thereafter, the optimized molecule in the QM part was extracted from the ONIOM model for the following single-point DFT calculations. All the quantum chemical calculations were conducted using the Gaussian 16 software package.[10]

5. Results and discussion

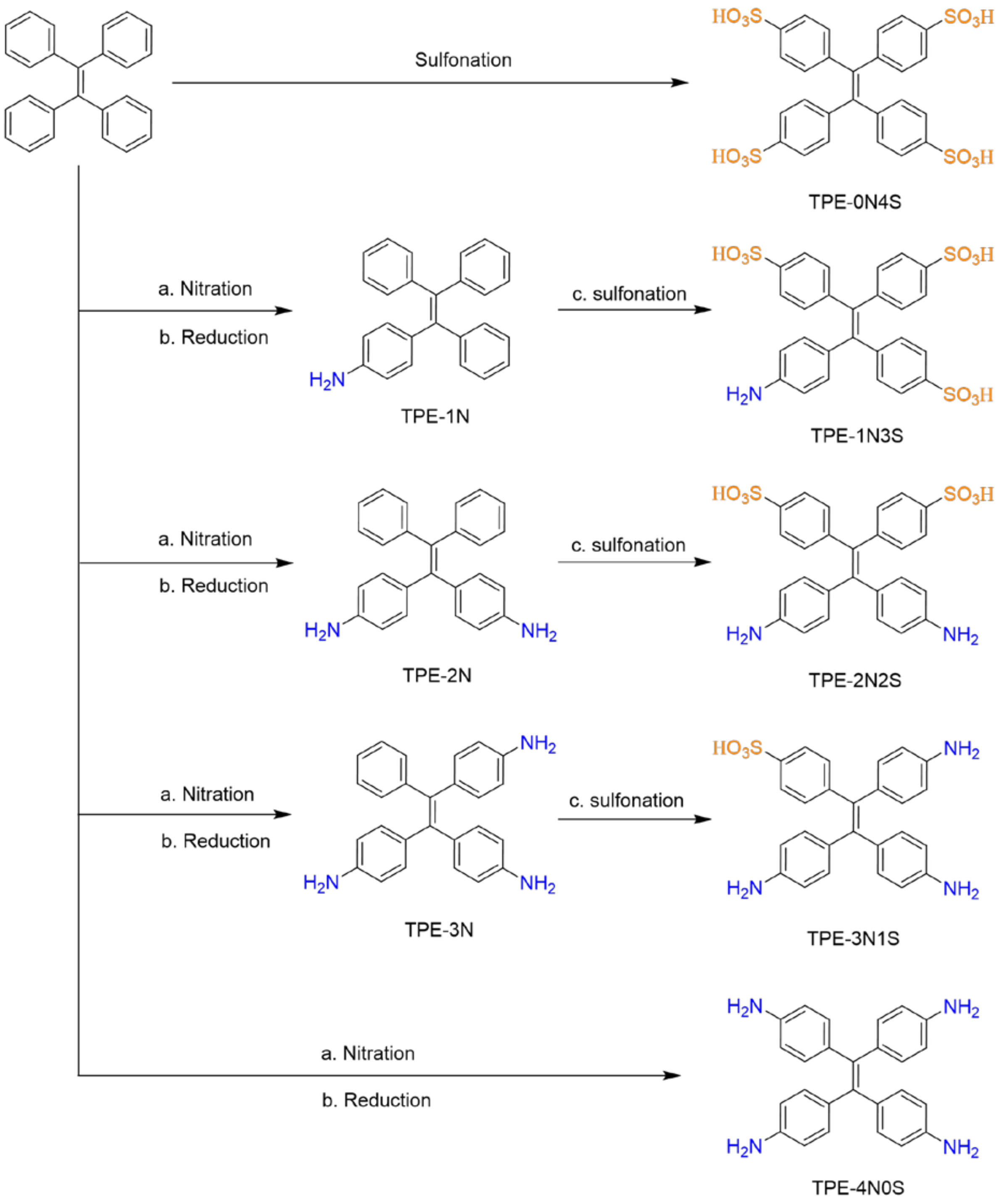

Figure S1 The synthetic route for chemicals.

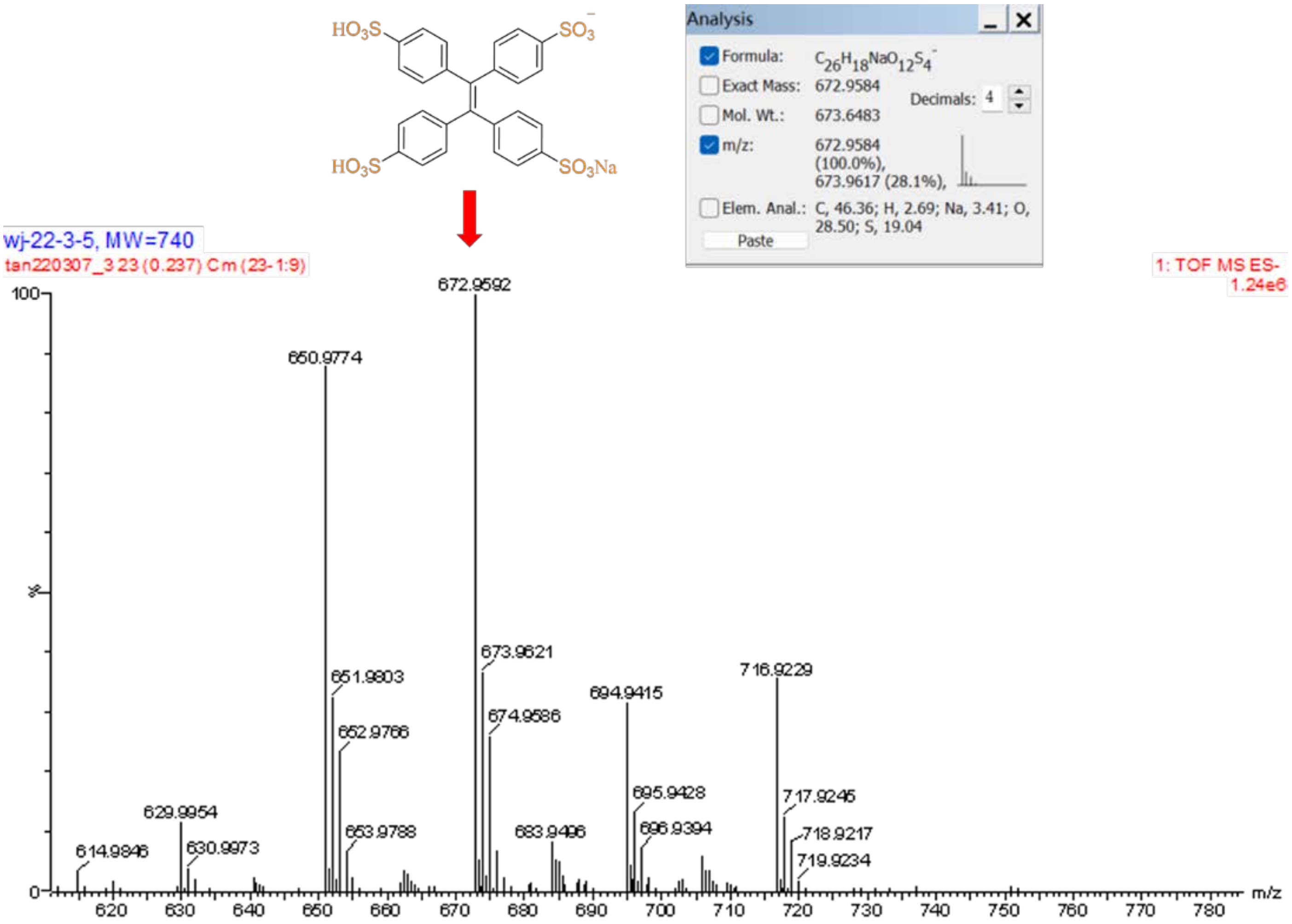

Figure S2a The HRMS spectral of TPE-0N4SNa.

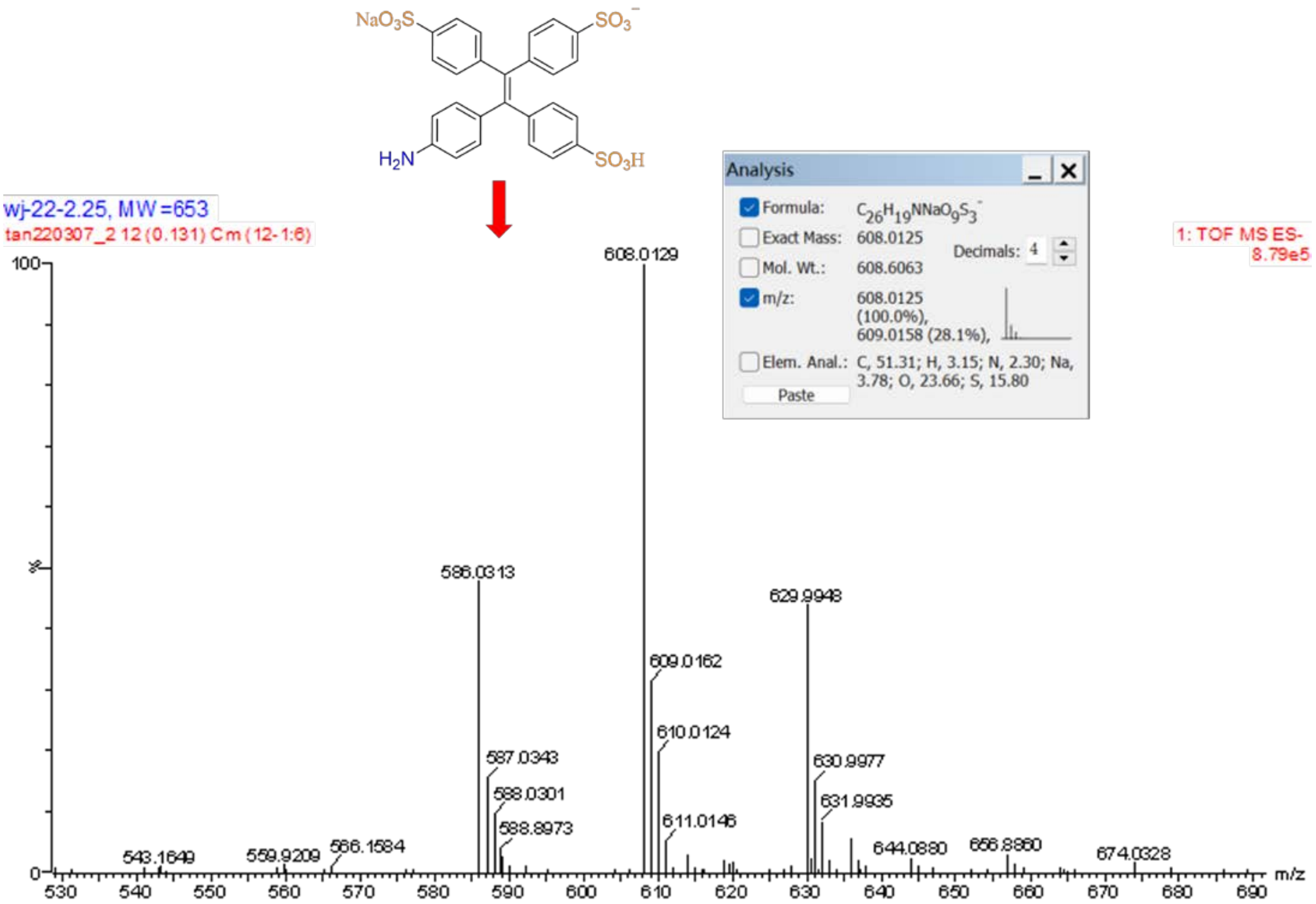

Figure S2b The HRMS spectral of TPE-1N3SNa.

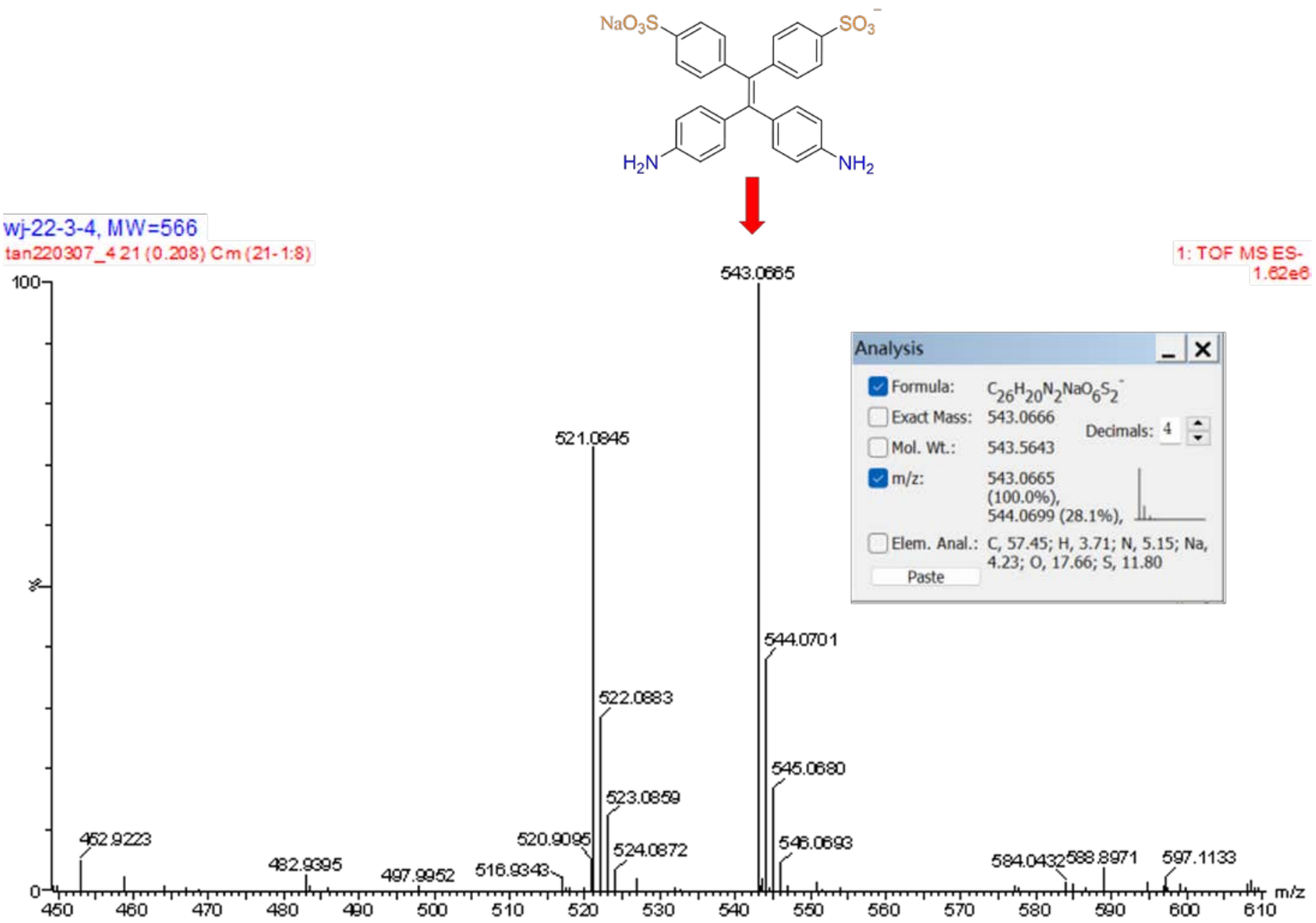

Figure S2c The HRMS spectral of TPE-2N2SNa.

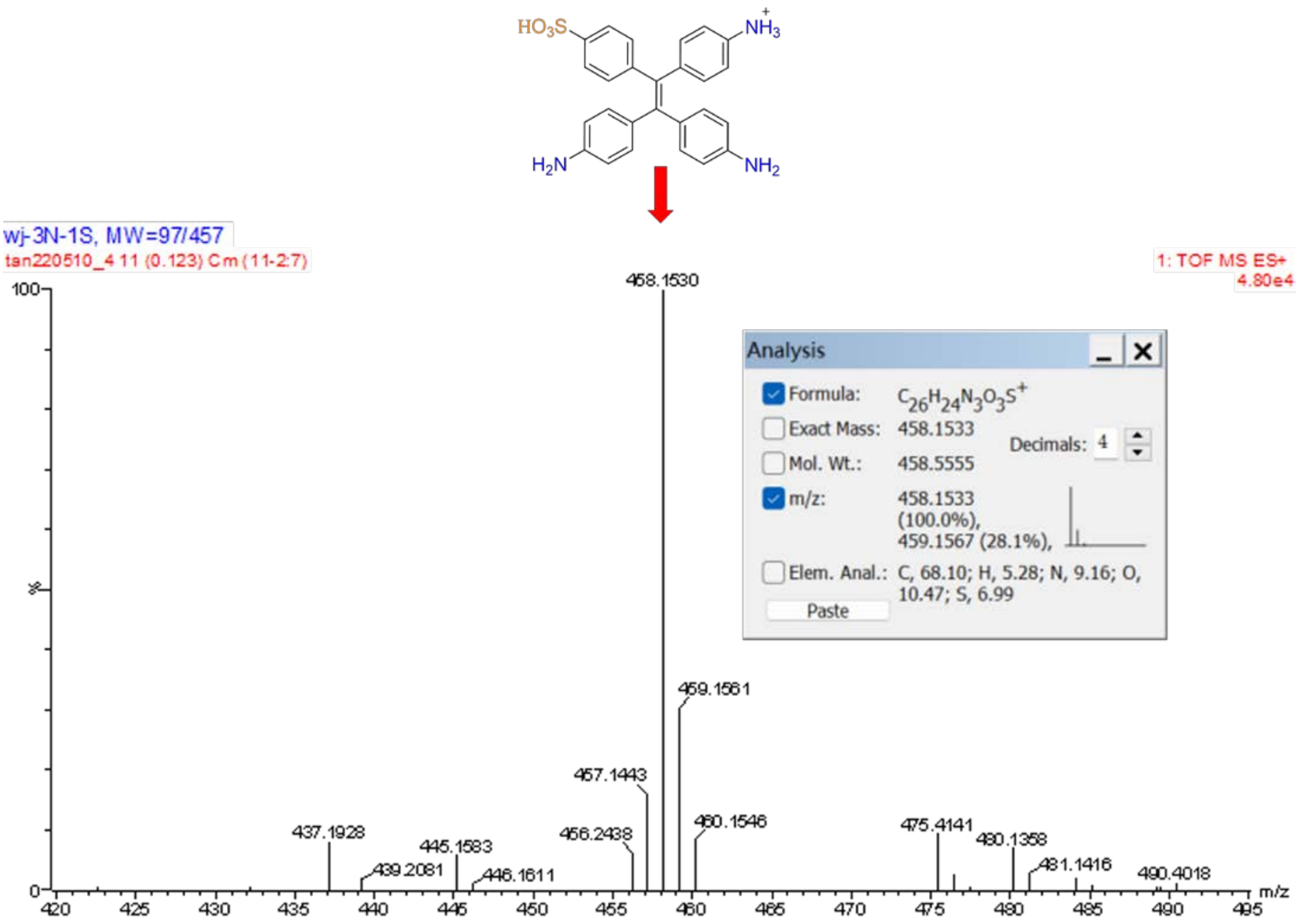

Figure S2d The HRMS spectral of TPE-3N1S.

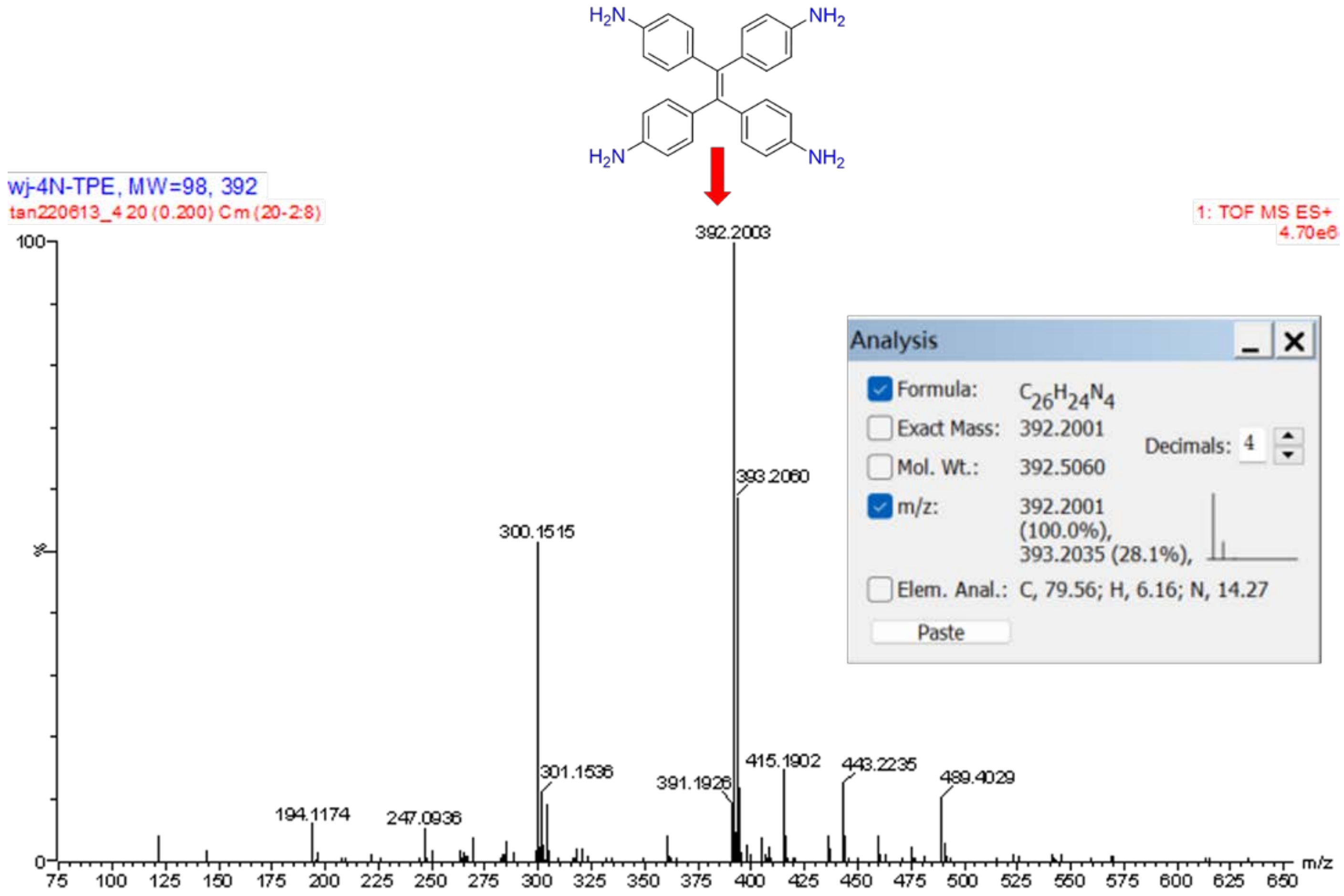

Figure S2e The HRMS spectral of TPE-4N0S.

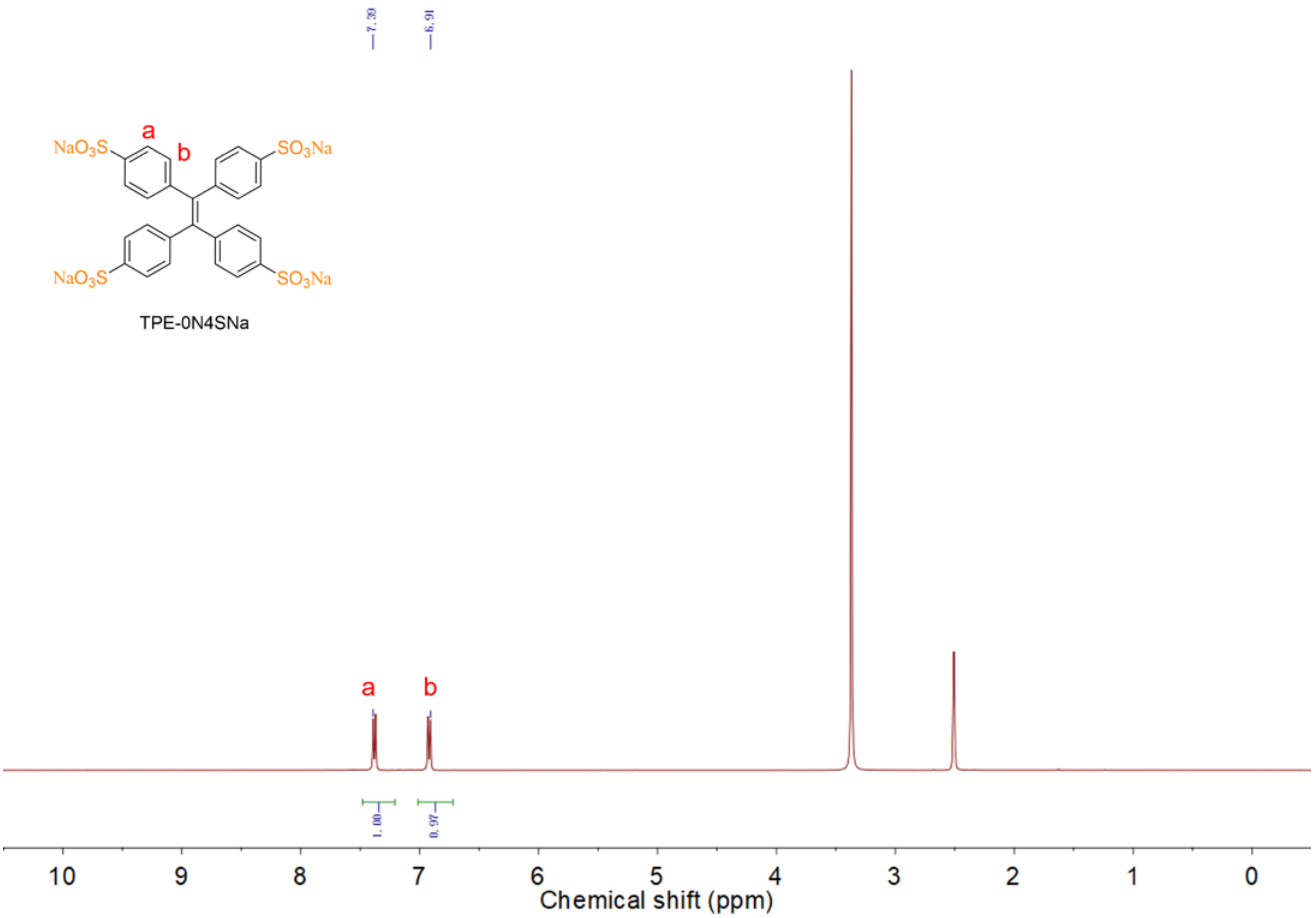

Figure S3a The ^{1}H-NMR spectral of TPE-0N4SNa in D-DMSO.

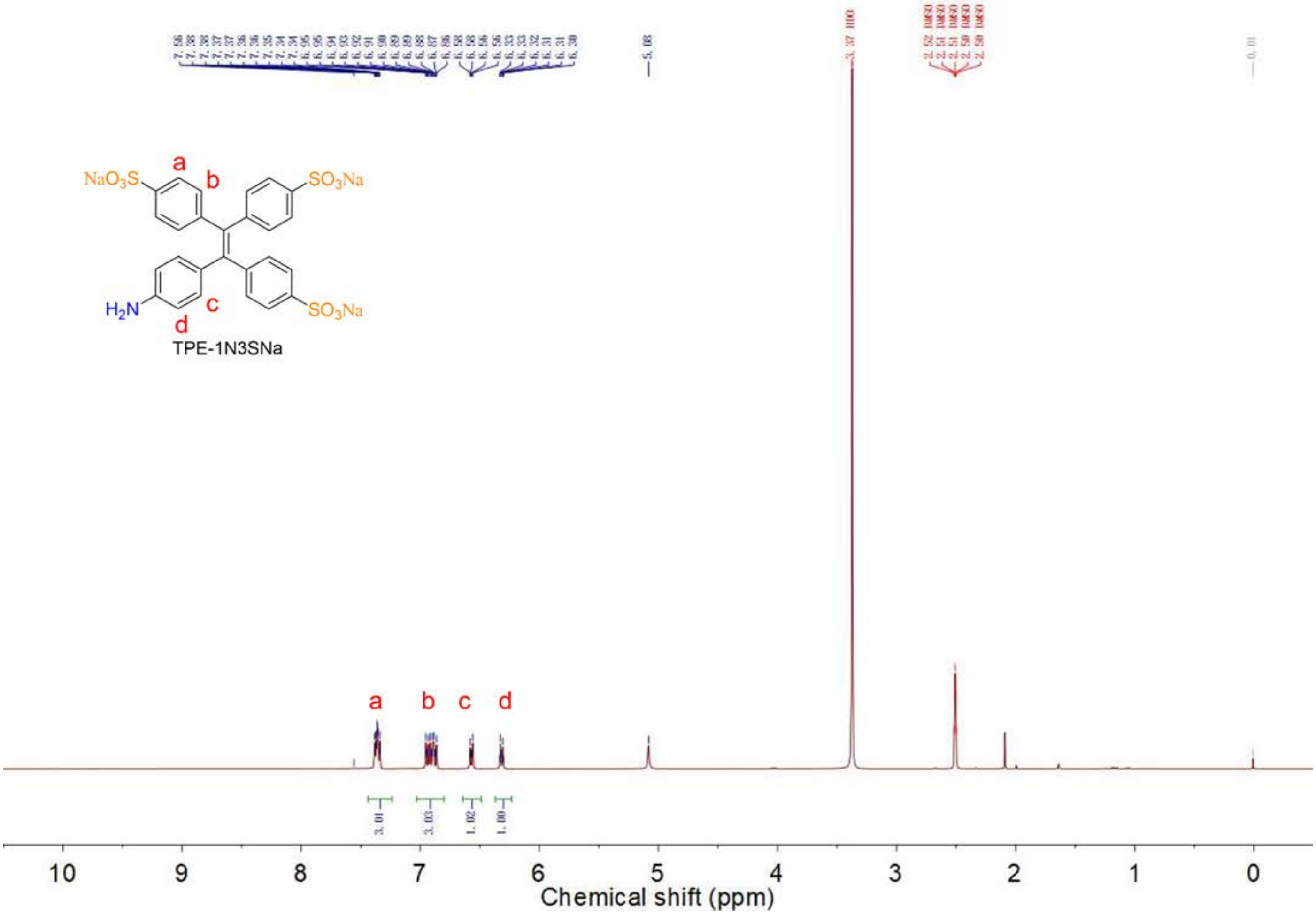

Figure S3b The ^{1}H-NMR spectral of TPE-1N3SNa in D-DMSO.

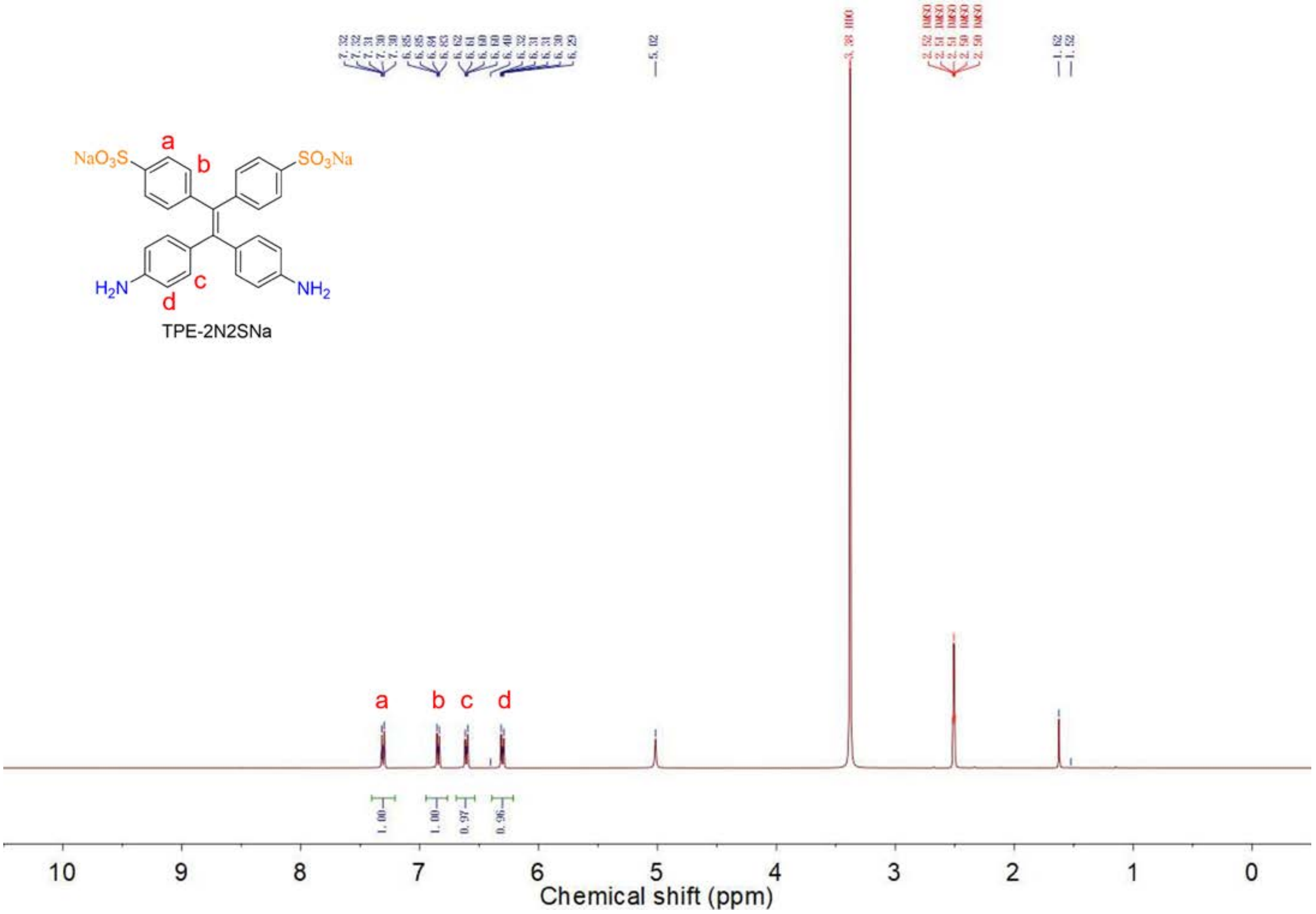

Figure S3c The ^{1}H-NMR spectral of TPE-2N2SNa in D-DMSO.

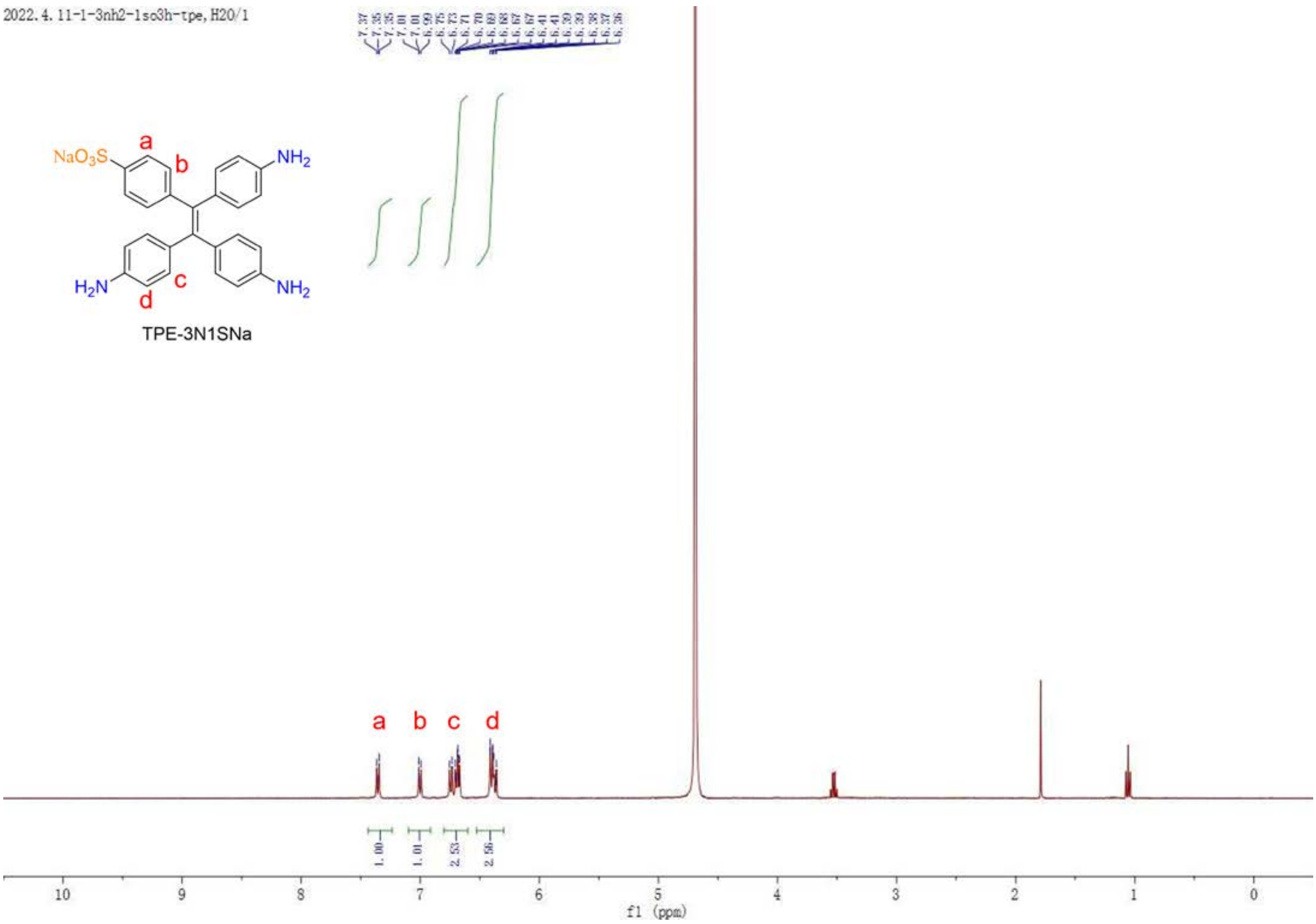

Figure S3d The ^{1}H-NMR spectral of TPE-3N1SNa in D_2O.

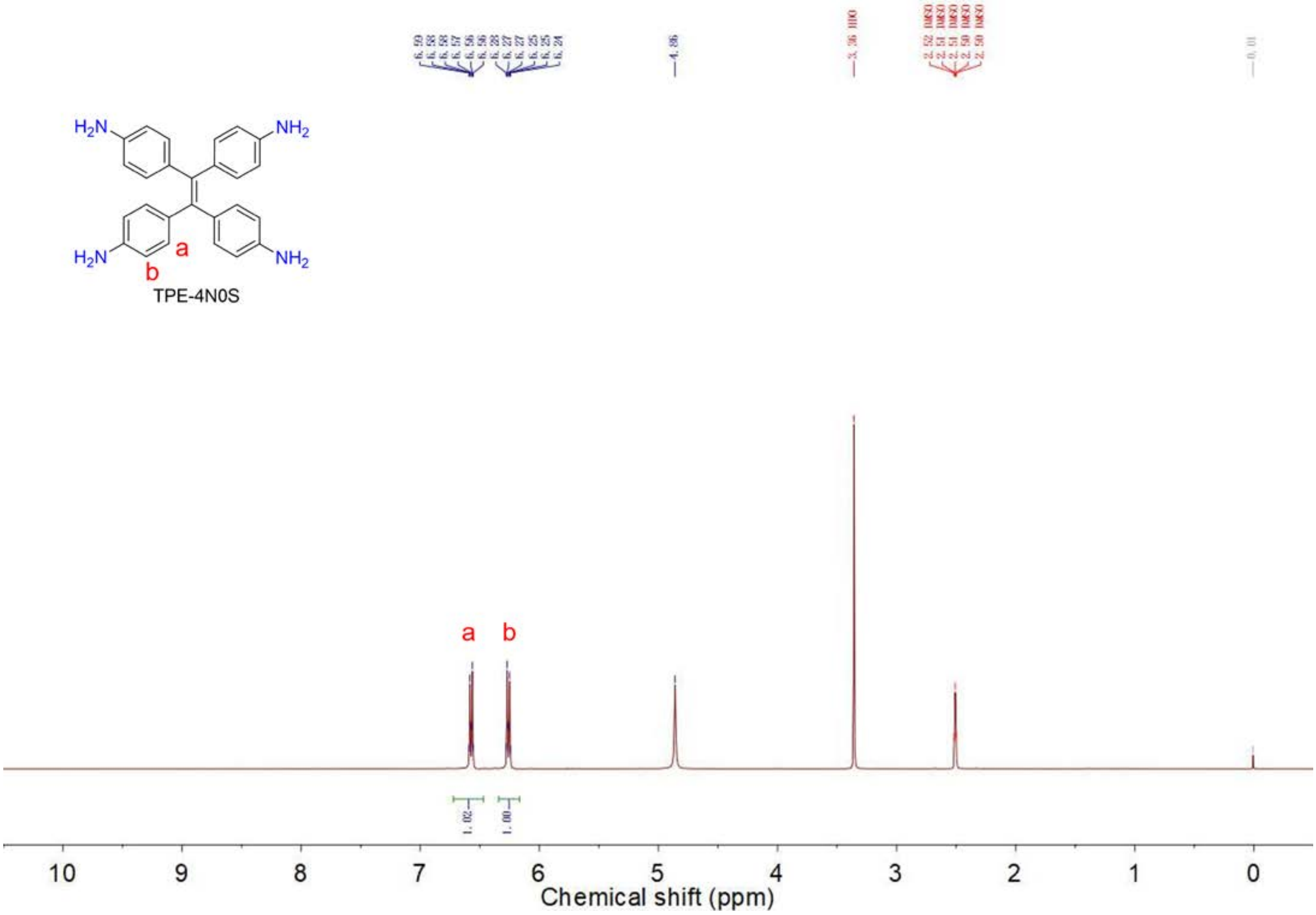

Figure S3e The ^{1}H-NMR spectral of TPE-4N0S in D-DMSO.

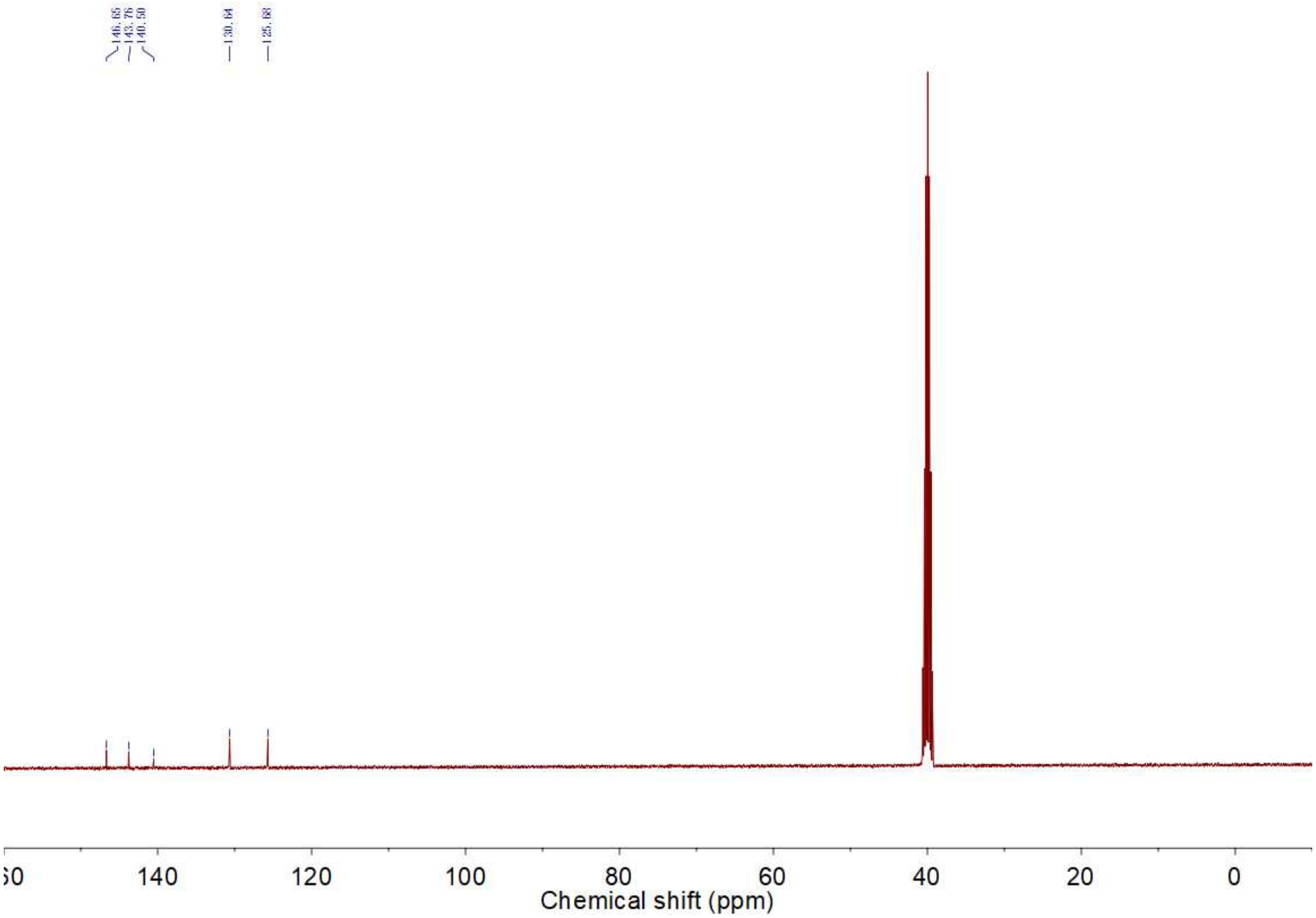

Figure S4a The ^{13}C-NMR spectral of TPE-0N4SNa in D-DMSO.

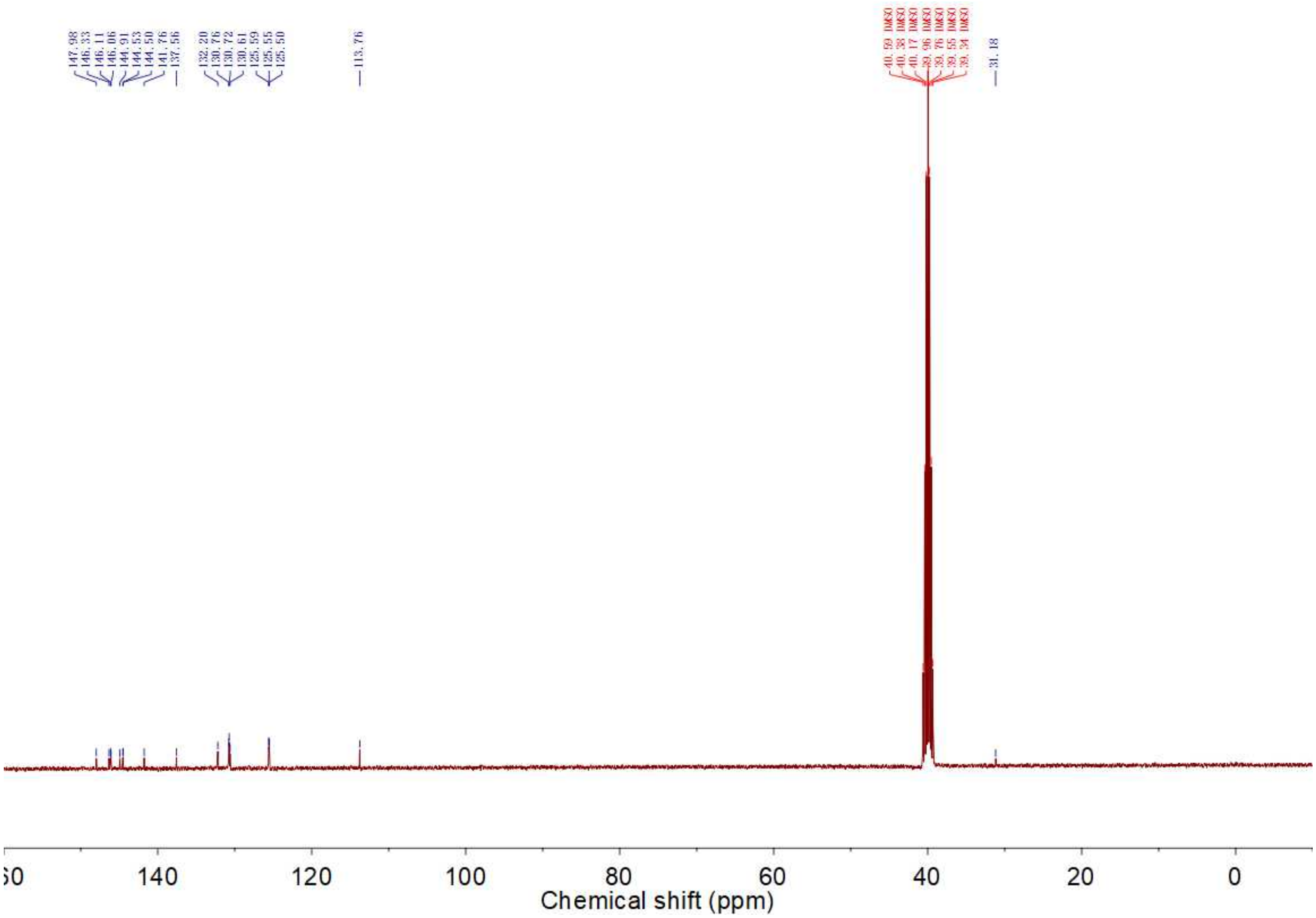

Figure S4b The ^{13}C-NMR spectral of TPE-1N3SNa in D-DMSO.

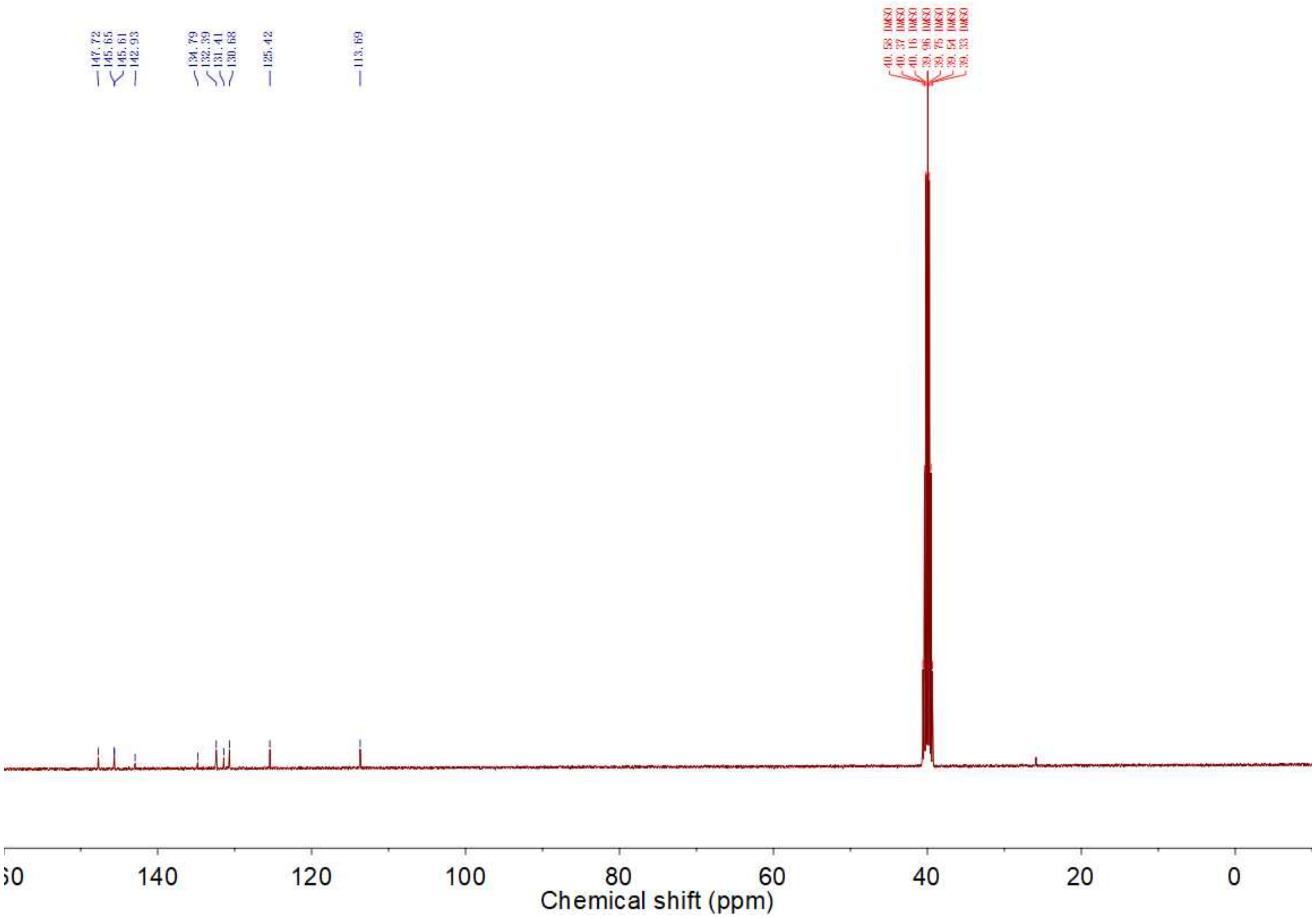

Figure S4c The ^{13}C-NMR spectral of TPE-2N2SNa in D-DMSO.

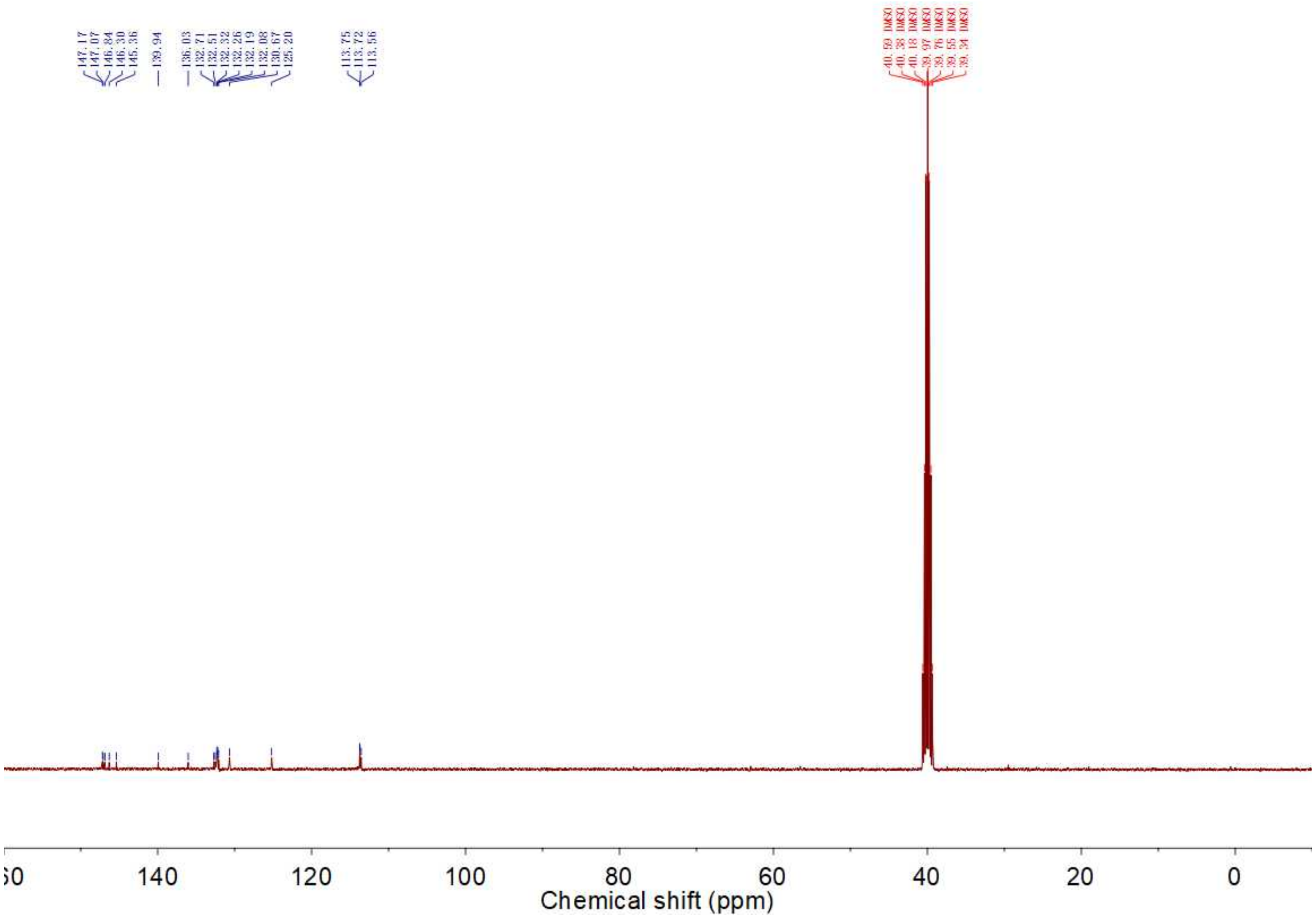

Figure S4d The ^{13}C-NMR spectral of TPE-3N1SNa in D-DMSO.

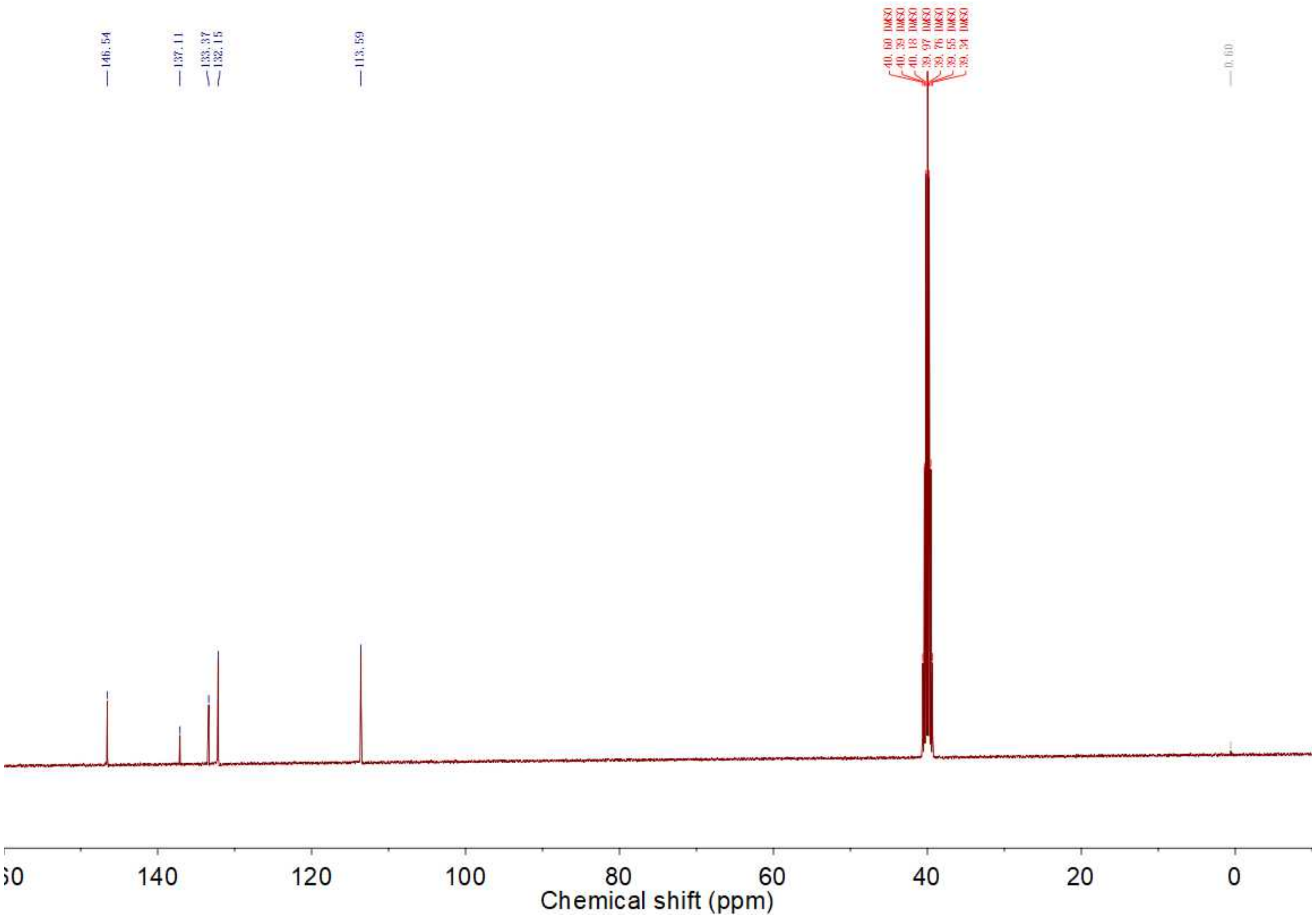

Figure S4e The ^{13}C-NMR spectral of TPE-4N0S in D-DMSO.

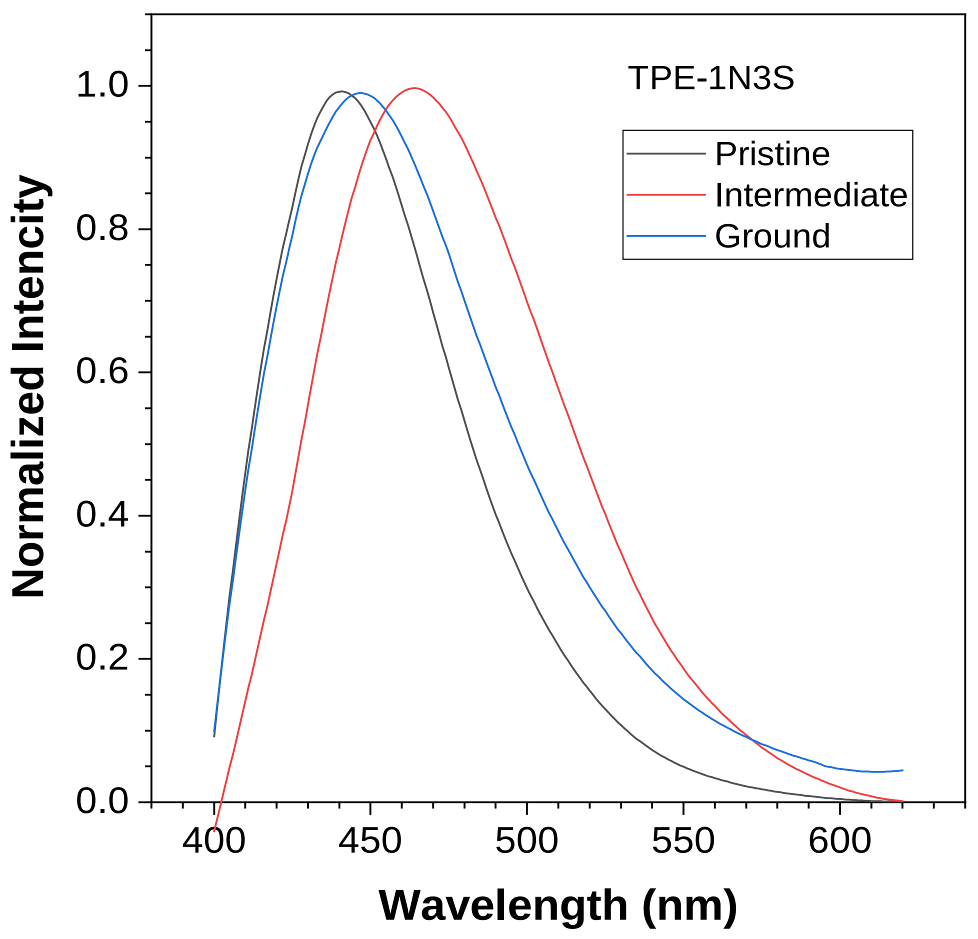

Figure S5 The PL spectral of TPE-IN3S at pristine powder, intermediate and ground states, respectively (excited at 365 nm).

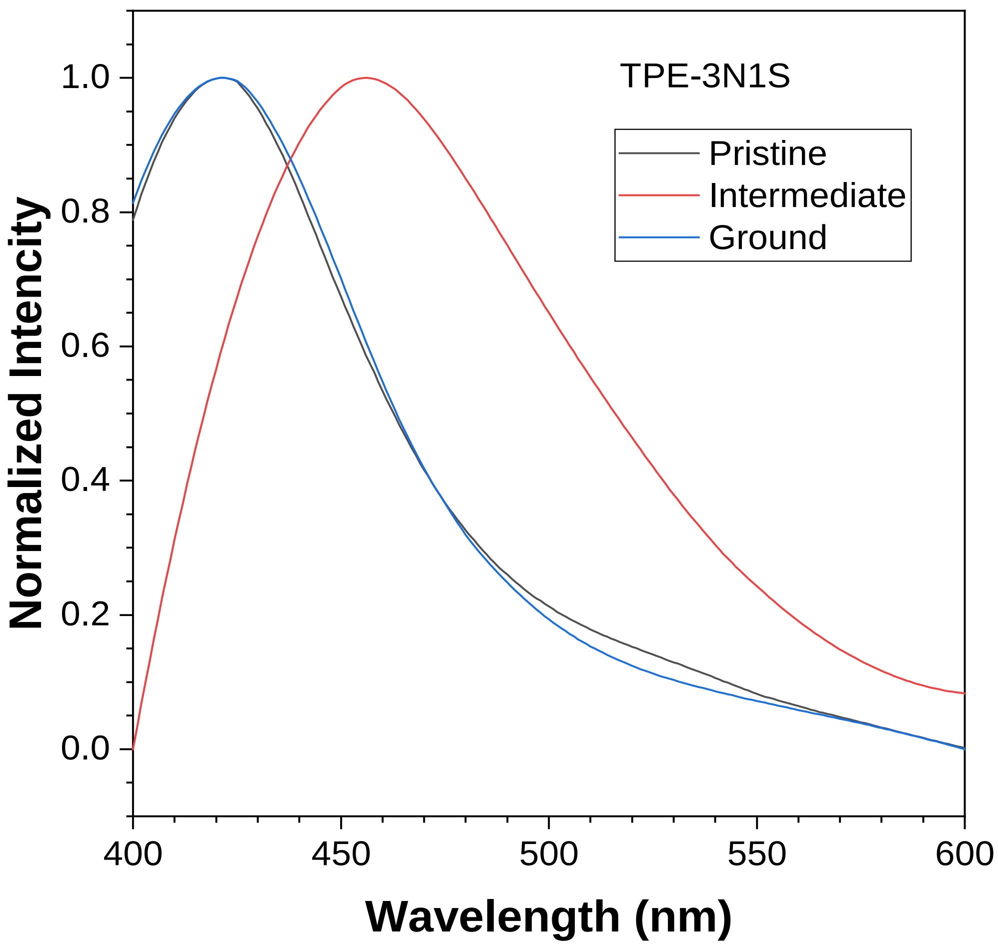

Figure S6 The PL spectral of TPE-3N1S at pristine powder, intermediate and ground states, respectively (excited at 365 nm).

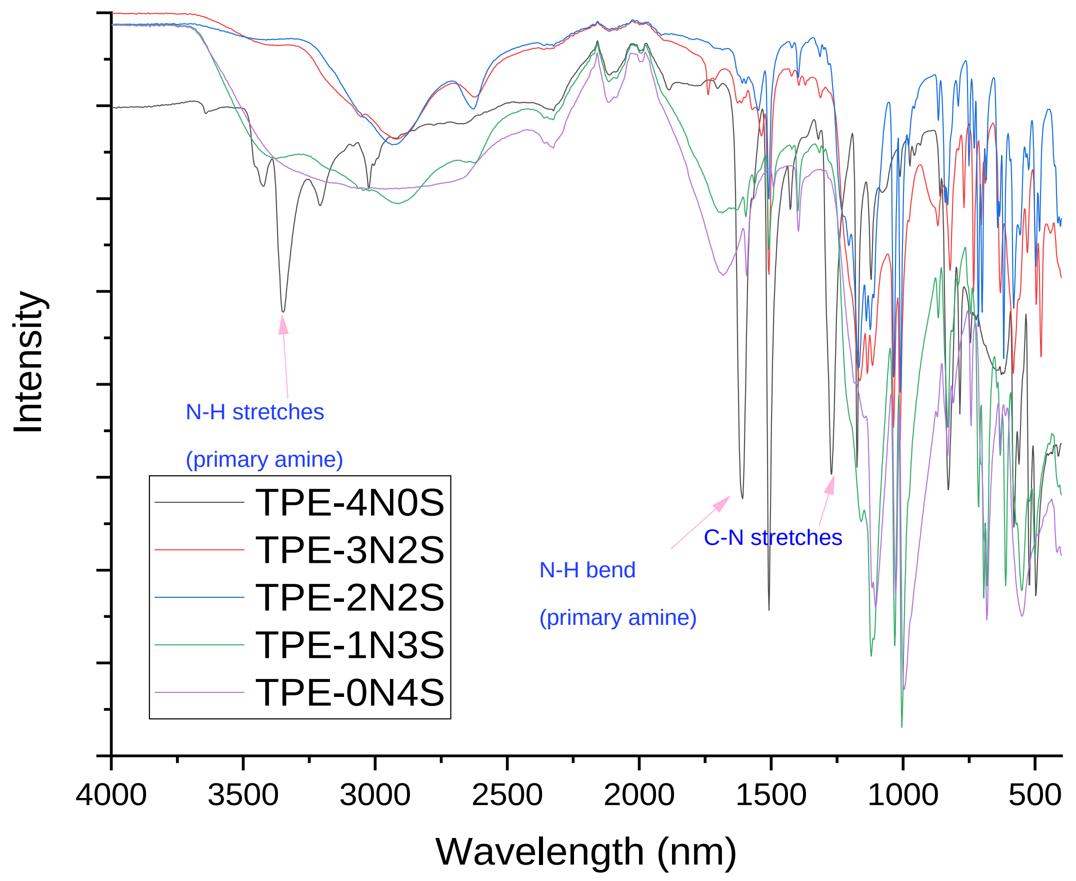

Figure S7 The FT-IR spectra of chemicals in pristine powder state.

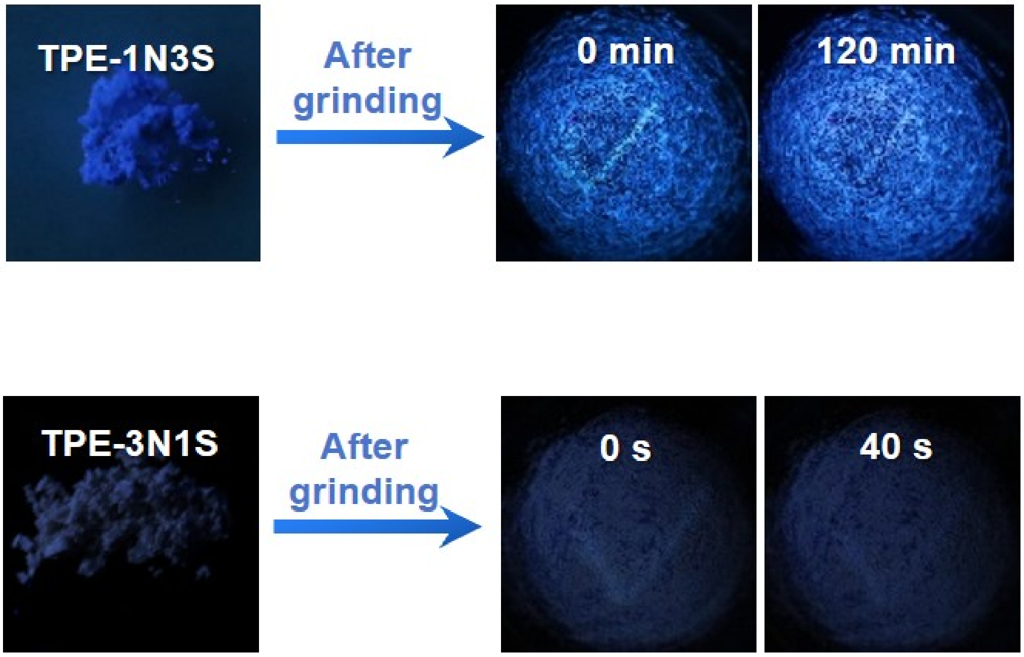

Figure S8 The images of TPE-1N3S and TPE-3N1S before and after scratching in UV light.

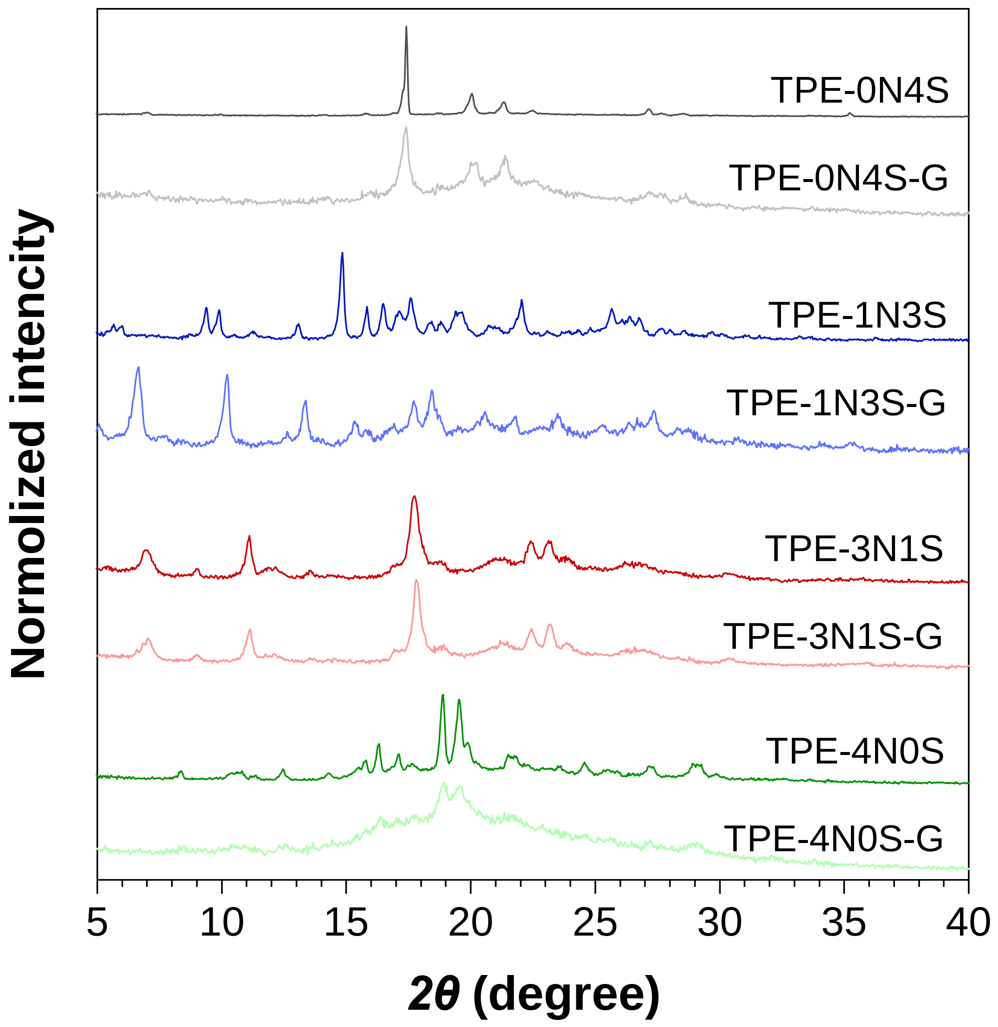

Figure S9 The PXRD patterns of chemicals before and after grinding.

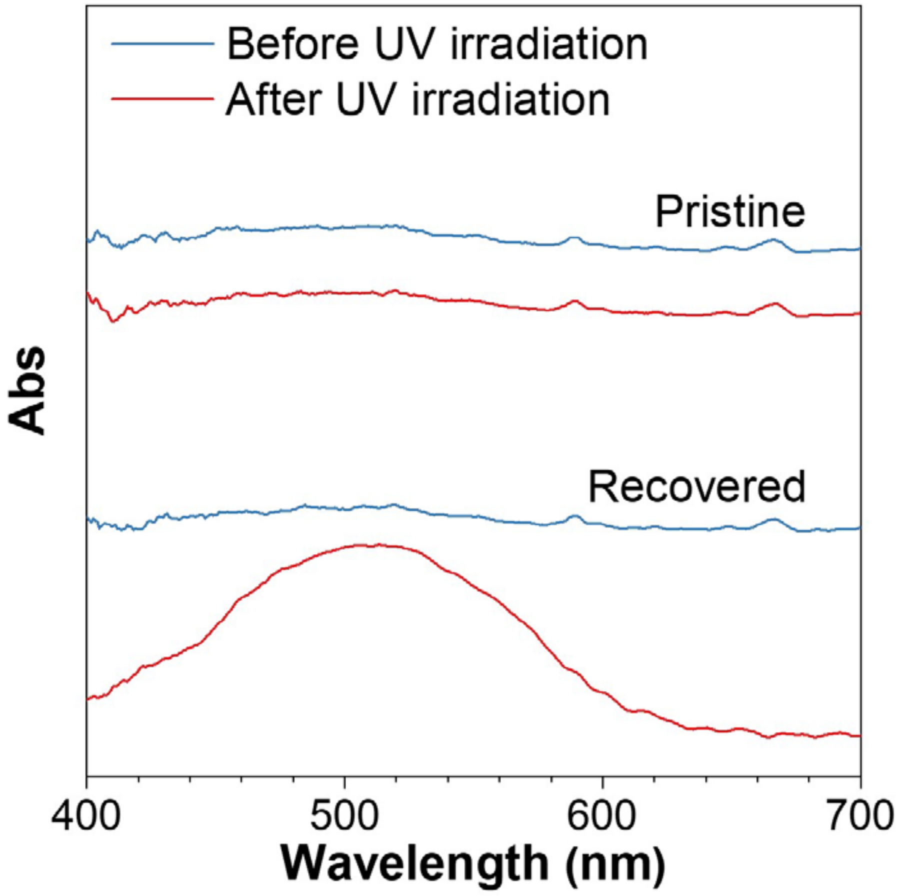

Figure S10 The absorption spectra of TPE-2N2S at pristine state and recovered state before and after UV irradiation.

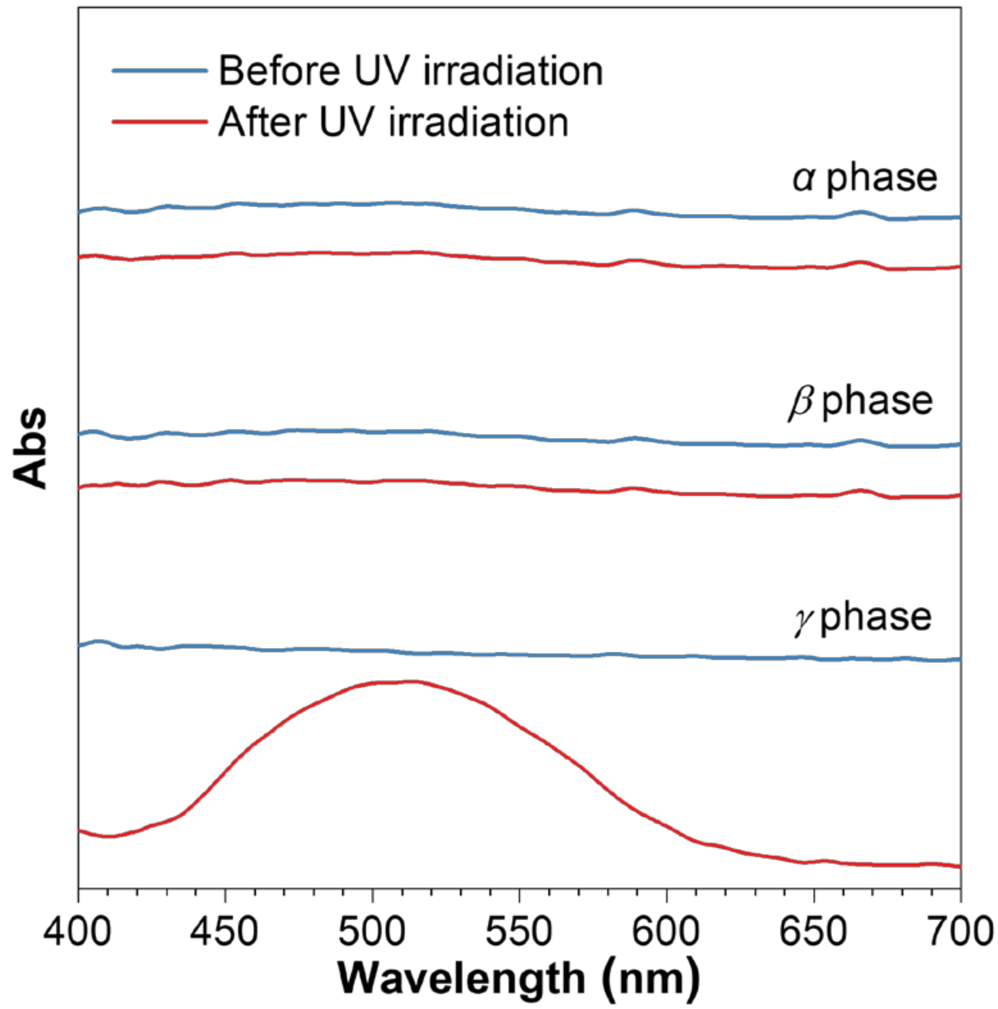

Figure S11 The absorption spectra of TPE-2N2S in different crystal phases before and after UV irradiation.

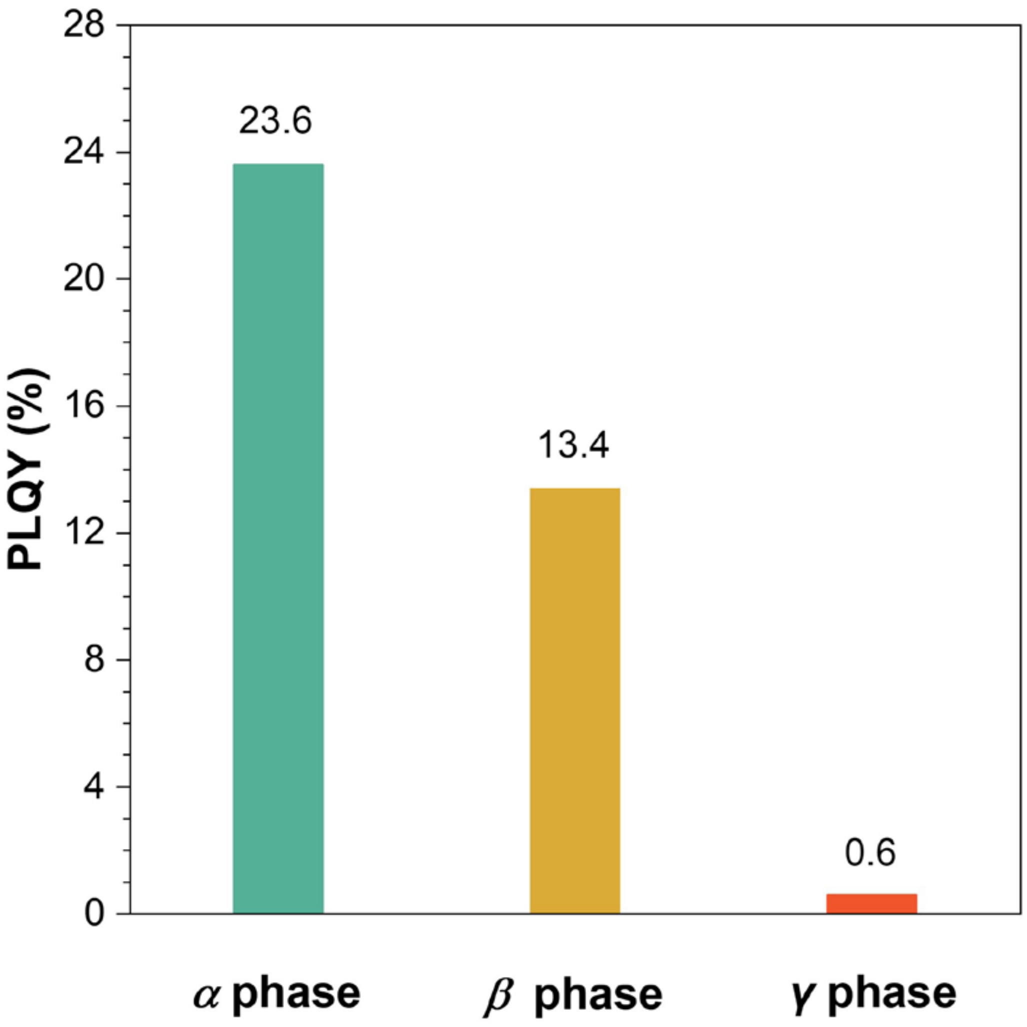

Figure S12 The PLQY values of TPE-2N2S in different crystalline phases.

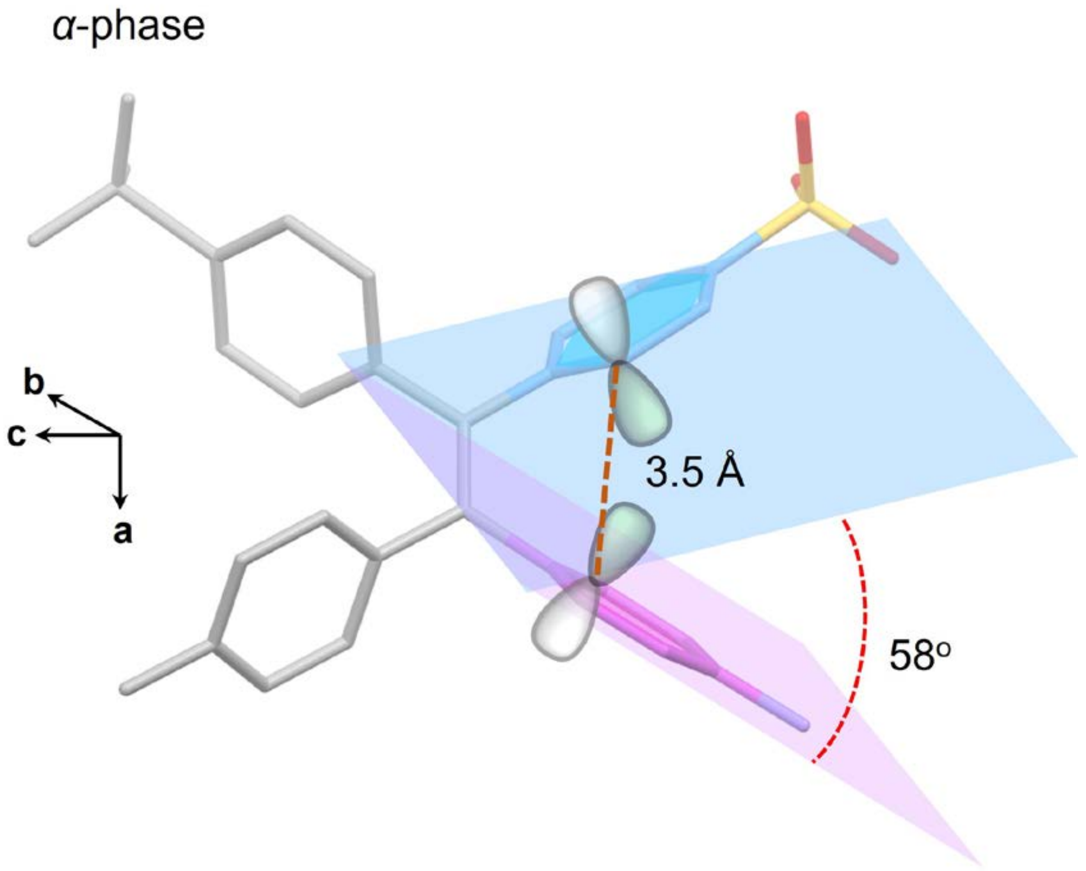

Figure S13 The single-molecular conformation of TPE-2N2S in crystalline α phase.

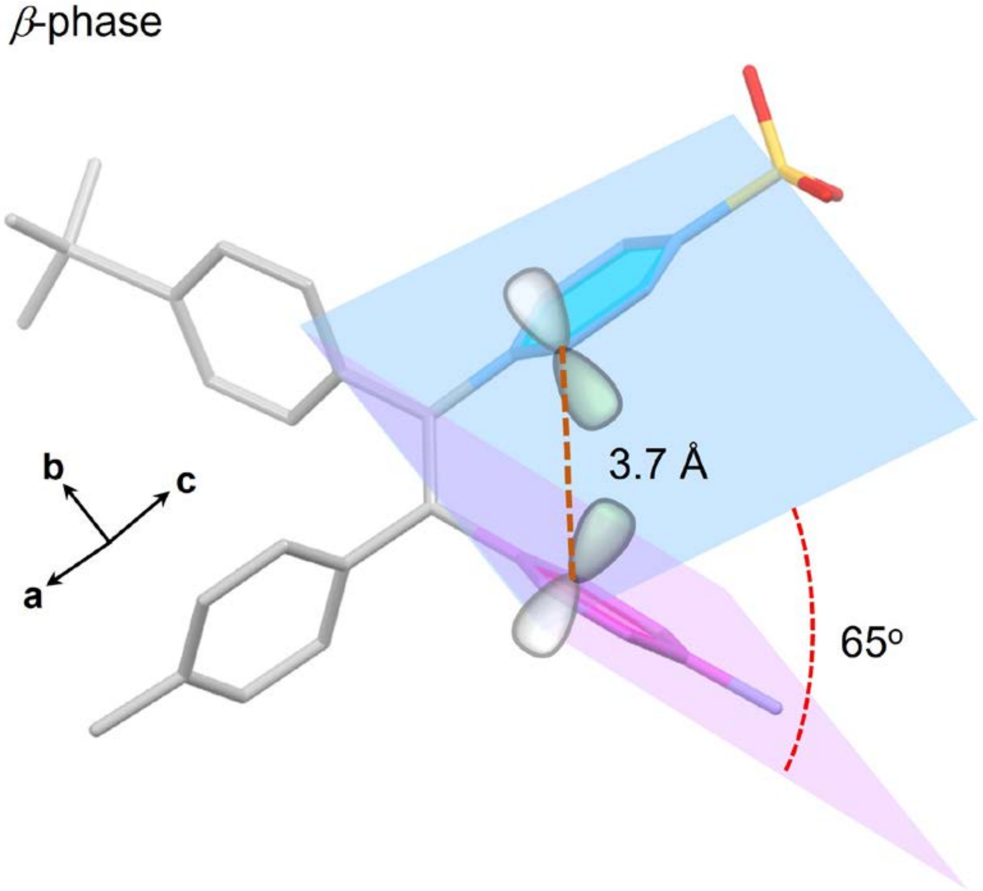

Figure S14 The single-molecular conformation of TPE-2N2S in crystalline β phase.

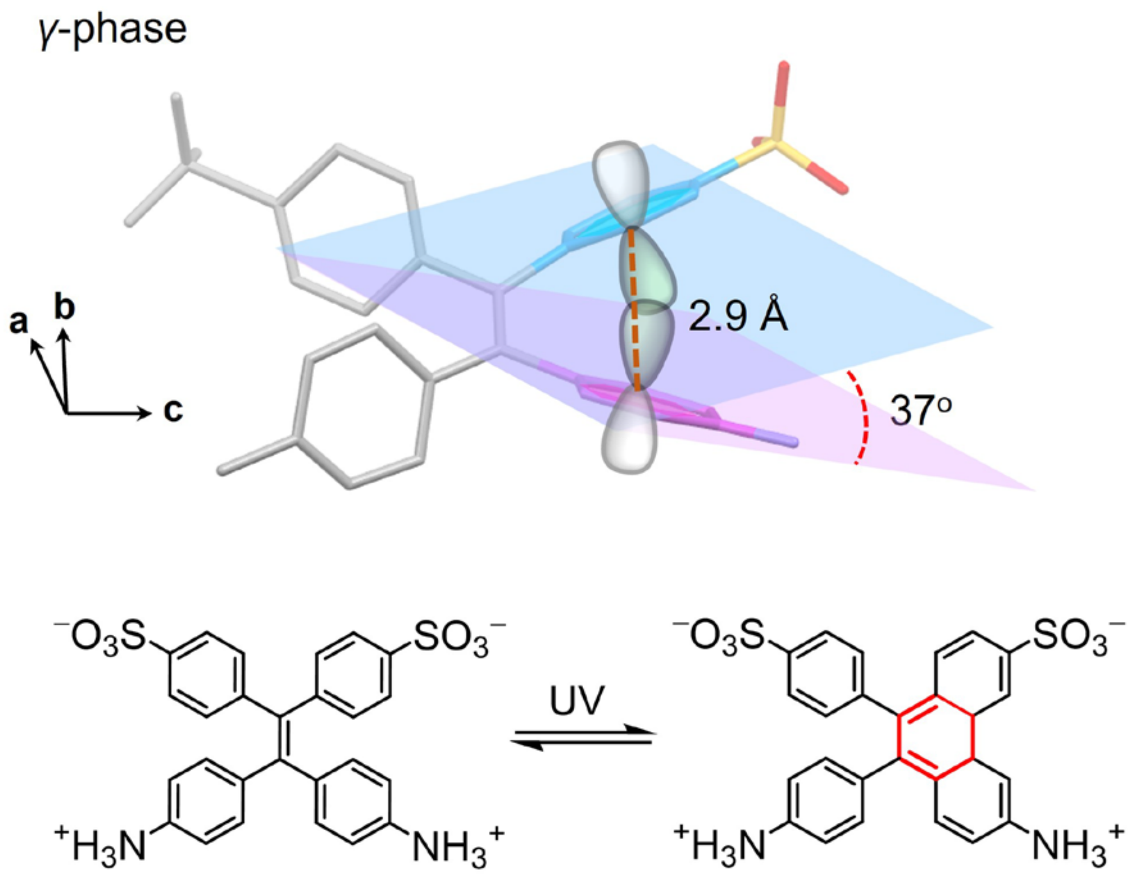

Figure S15 The single-molecular conformation of TPE-2N2S in crystalline γ phase. Inset: the photocyclization reaction of TPE-2N2S.

Table S1 Crystal data and structure refinement for crystal-*β* of TPE-2N2S.

Identification code	wangjin21CuLT_auto
Empirical formula	$C_{34}H_{46}N_2O_{10}S_6$
Formula weight	835.09
Temperature/K	100.01(10)
Crystal system	tetragonal
Space group	$I4_1cd$
a/Å	19.5421(2)
b/Å	19.5421(2)
c/Å	20.9646(3)
α/°	90
β/°	90
γ/°	90
Volume/Å^3	8006.2(2)
Z	8
ρ_{calc}g/cm^3	1.386
μ/mm^{-1}	3.626
F(000)	3520.0
Crystal size/mm^3	0.12 × 0.04 × 0.04
Radiation	Cu Kα (λ = 1.54184)
2Θ range for data collection/°	9.05 to 148.786
Index ranges	$-21 \le h \le 24$, $-23 \le k \le 24$, $-26 \le l \le 25$
Reflections collected	22261
Independent reflections	4052 [R_{int} = 0.0498, R_{sigma} = 0.0293]
Data/restraints/parameters	4052/79/280
Goodness-of-fit on F^2	1.060
Final R indexes [I>=2σ (I)]	R_1 = 0.0437, wR_2 = 0.1164
Final R indexes [all data]	R_1 = 0.0470, wR_2 = 0.1201
Largest diff. peak/hole / e Å^{-3}	0.60/-0.29
Flack parameter	-0.004(17)

Crystal structure data can be obtained free of charge from the Cambridge Crystallographic Data Centre via https://www.ccdc.cam.ac.uk and have been allocated the accession number CCDC 2354896.

Table S2 Crystal data and structure refinement for TPE-0N4S.

Identification code	4s_0m_sq_sq
Empirical formula	$C_{39}H_{32}O_{22}S_6$
Formula weight	1045.00
Temperature/K	100.0
Crystal system	cubic
Space group	Pn-3
a/Å	18.7785(2)
b/Å	18.7785(2)
c/Å	18.7785(2)
α/°	90
β/°	90
γ/°	90
Volume/Å3	6621.9(2)
Z	4
ρcalcg/cm3	1.048
μ/mm-1	2.418
F(000)	2152.0
Crystal size/mm3	0.14 × 0.13 × 0.12
Radiation	CuKα (λ = 1.54178)
2Θ range for data collection/°	6.656 to 126.94
Index ranges	$-18 \le h \le 14$, $-21 \le k \le 21$, $-16 \le l \le 21$
Reflections collected	17729
Independent reflections	1794 [Rint = 0.1064, Rsigma = 0.0374]
Data/restraints/parameters	1794/54/110
Goodness-of-fit on F2	1.078
Final R indexes [I>=2σ (I)]	R1 = 0.1070, wR2 = 0.2756
Final R indexes [all data]	R1 = 0.1482, wR2 = 0.3366
Largest diff. peak/hole / e Å-3	0.58/-0.45

Crystal structure data can be obtained free of charge from the Cambridge Crystallographic Data Centre via https://www.ccdc.cam.ac.uk and have been allocated the accession number CCDC 2355139.

Table S3 Crystal data and structure refinement for TPE-4N0S.

Identification code	wangjin25CuLT_auto
Empirical formula	$C_{78}H_{73.5}N_{12}O_{0.75}$
Formula weight	1190.98
Temperature/K	100.00(10)
Crystal system	monoclinic
Space group	$P2_1/c$
a/Å	32.2605(5)
b/Å	9.20827(17)
c/Å	21.4765(4)
α/°	90
β/°	98.9550(17)
γ/°	90
Volume/Å^3	6302.1(2)
Z	4
ρ_{calc}g/cm^3	1.255
μ/mm^{-1}	0.594
F(000)	2526.0
Crystal size/mm^3	0.2 × 0.08 × 0.05
Radiation	Cu Kα (λ = 1.54184)
2Θ range for data collection/°	5.546 to 149.224
Index ranges	$-40 \leq h \leq 31$, $-11 \leq k \leq 10$, $-24 \leq l \leq 26$
Reflections collected	21901
Independent reflections	12313 [R_{int} = 0.0538, R_{sigma} = 0.0707]
Data/restraints/parameters	12313/176/907
Goodness-of-fit on F^2	1.036
Final R indexes [I>=2σ (I)]	R_1 = 0.0544, wR_2 = 0.1296
Final R indexes [all data]	R_1 = 0.0811, wR_2 = 0.1493
Largest diff. peak/hole / e Å^{-3}	0.31/-0.36

Crystal structure data can be obtained free of charge from the Cambridge Crystallographic Data Centre via https://www.ccdc.cam.ac.uk and have been allocated the accession number CCDC 2354897.

5. Supplementary references

S1. Vanommeslaeghe, K.; Raman, E. P.; MacKerell, A. D., Jr., Automation of the CHARMM General Force Field (CGenFF) II: assignment of bonded parameters and partial atomic charges. *Journal of chemical information and modeling* **2012,** *52* (12), 3155-3168.

S2. Vanommeslaeghe, K.; MacKerell, A. D., Jr., Automation of the CHARMM General Force Field (CGenFF) I: bond perception and atom typing. *Journal of chemical information and modeling* **2012,** *52* (12), 3144-3154.

S3. Bussi, G.; Donadio, D.; Parrinello, M., Canonical sampling through velocity rescaling. *J Chem Phys* **2007,** *126* (1), 014101.

S4. Berendsen, H. J. C.; Postma, J. P. M.; van Gunsteren, W. F.; DiNola, A.; Haak, J. R., Molecular dynamics with coupling to an external bath. *The Journal of Chemical Physics* **1984,** *81* (8), 3684-3690.

S5. Essmann, U.; Perera, L.; Berkowitz, M. L.; Darden, T.; Lee, H.; Pedersen, L. G., A smooth particle mesh Ewald method. *The Journal of Chemical Physics* **1995,** *103* (19), 8577-8593.

S6. Darden, T.; York, D.; Pedersen, L., Particle mesh Ewald: An Nlog(N) method for Ewald sums in large systems. *The Journal of Chemical Physics* **1993,** *98* (12), 10089-10092.

S7. Hess, B.; Bekker, H.; Berendsen, H. J. C.; Fraaije, J. G. E. M., LINCS: A linear constraint solver for molecular simulations. *Journal of Computational Chemistry* **1997,** *18* (12), 1463-1472.

S8. Dolan, E. A.; Venable, R. M.; Pastor, R. W.; Brooks, B. R., Simulations of Membranes and Other Interfacial Systems Using P21 and Pc Periodic Boundary Conditions. *Biophysical Journal* **2002,** *82* (5), 2317-2325.

S9. Abraham, M. J.; Murtola, T.; Schulz, R.; Páll, S.; Smith, J. C.; Hess, B.; Lindahl, E., GROMACS: High performance molecular simulations through multi-level parallelism from laptops to supercomputers. *SoftwareX* **2015,** *1-2*, 19-25.

S10. Frisch, M. J., Trucks, G.W., Schlegel, H.B., Scuseria, G.E., Robb, M.A., Cheeseman, J.R., Scalmani, G., Barone, V., Petersson, G.A., Nakatsuji, H., Li, X., Caricato, M., Marenich, A.V., Bloino, J., Janesko, B.G., Gomperts, R., Mennucci, B., Hratchian, H.P., Ortiz, J.V., Izmaylov, A.F., Sonnenberg, J.L., Williams-Young, D., Ding, F., Lipparini, F., Egidi, F., Goings, J., Peng, B., Petrone, A., Henderson, T., Ranasinghe, D., Zakrzewski, V.G., Gao, J., Rega, N., Zheng, G., Liang, W., Hada, M., Ehara, M., Toyota, K., Fukuda, R., Hasegawa, J., Ishida, M., Nakajima, T., Honda, Y., Kitao, O., Nakai, H., Vreven, T., Throssell, K., Montgomery, Jr., J.A., Peralta, J.E., Ogliaro, F., Bearpark, M.J., Heyd, J.J., Brothers, E.N., Kudin, K.N., Staroverov, V.N., Keith, T.A., Kobayashi, R., Normand, J., Raghavachari, K., Rendell, A.P., Burant, J.C., Iyengar, S.S., Tomasi, J., Cossi, M., Millam, J.M., Klene, M., Adamo, C., Cammi, R., Ochterski, J.W., Martin, R.L., Morokuma, K., Farkas, O., Foresman, J.B. and Fox, D.J., Gaussian 16, Revision A. 03. Gaussian, Inc., Wallingford CT. **2016**.
